# Global characterization of GH11 family xylanases genes in *Neostagonosporella sichuanensis* and functional analysis of *Nsxyn1* and *Nsxyn2*

**DOI:** 10.3389/fmicb.2024.1507998

**Published:** 2024-11-21

**Authors:** Lijuan Liu, Chengsong Li, Fang Liang, Shan Han, Shujiang Li, Chunlin Yang, Yinggao Liu

**Affiliations:** ^1^College of Forestry, Sichuan Agricultural University, Chengdu, China; ^2^National Forestry and Grassland Administration Key Laboratory of Forest Resources Conservation and Ecological Safety on the Upper Reaches of the Yangtze River and Forestry Ecological Engineering in the Upper Reaches of the Yangtze River Key Laboratory of Sichuan Province, College of Forestry, Sichuan Agricultural University, Chengdu, China

**Keywords:** *Neostagonosporella sichuanensis*, xylanase, prokaryotic expression, transient expression, gene knockout

## Abstract

Rhombic-spot disease, caused mainly by *Neostagonosporella sichuanensis*, significantly impacts the yield and quality of fishscale bamboo (*Phyllostachys heteroclada*). Xylanases are essential for pathogenic fungi infection, yet their specific functions in the physiology and pathogenicity of *N. sichuanensis* remain unclear. Here, we characterized three xylanase proteins with glycosyl hydrolase 11 domains from the *N. sichuanensis* SICAUCC 16–0001 genome and examined the function of Nsxyn1 and Nsxyn2. Purified Nsxyn1 and Nsxyn2 proteins displayed specific xylanase activity *in vitro* and induced cell death in *Nicotiana benthamiana*, independent of their enzymatic function. Both proteins possessed signal peptides and were confirmed as secreted proteins using a yeast secretion system. Subcellular localization revealed that Nsxyn1 and Nsxyn2 localized in both the cytoplasm and nucleus and can trigger cell death in *N. benthamiana* through *Agrobacterium tumefaciens*-mediated transient transformation. qRT-PCR results showed notable upregulation of *Nsxyn1* and *Nsxyn2* during infection, with *Nsxyn1* exhibiting an 80-fold increase at 15 days post-inoculation. Deletion of *Nsxyn1* and *Nsxyn2* in *N. sichuanensis* impaired xylan degradation, adaptation to osmotic and oxidative stress, and pathogenic full virulence. Deletion of *Nsxyn1* notably slowed fungal growth and reduced spore production, whereas only a reduction in microconidial production was observed in *Nsxyn2* mutants. Complementation of *Nsxyn1* and *Nsxyn2* only partially restored these phenotypic defects in the ∆*Nsxyn1* and ∆*Nsxyn2* mutants. These findings suggest that Nsxyn1 and Nsxyn2 contribute to *N. sichuanensis* virulence and induced plant defense responses, providing new insights into the function of xylanases in the interaction between fishscale bamboo and *N. sichuanensis*.

## 1 Introduction

Known for its rapid growth and sustainability, bamboo is an essential resource worldwide (especially in Asia). *Phyllostachys heteroclada* Oliver, known as fishscale bamboo, is a crucial waterlogging-tolerant and industrial bamboo species native to China. It plays an important role in riparian zone restoration, bamboo shoots, and handicraft production and serves as a vital food source for wild giant pandas (Jing et al., 2022; Liang et al., 2024; Shi and Chen, 2022). The culms are the primary economic component of bamboo; however, culm diseases significantly impact bamboo wood quality, leading to considerable ecological and financial losses (Huang L. et al., 2023). Although numerous culm diseases have been reported, there is a lack of in-depth studies (Huang L. et al., 2023; Yang et al., 2024). Notably, major culm diseases such as bamboo blight and bamboo witches’ broom are receiving increased attention, including investigations into their pathogenic molecular mechanisms (Li et al., 2020; Gu et al., 2024). For example, the whole genome of the bamboo blight pathogen, *Arthrinium phaeospermum*, has been published, identifying key pathogenic factors such as the *ApCE12* and *ApCE22* effectors and *Ctf1β* cutinase (Li et al., 2020; Fang et al., 2021; Fang et al., 2022). Among these diseases, rhombic-spot disease is recognized as one of the most significant threats to fishscale bamboo, with *Neostagonosporella sichuanensis* being the most commonly reported pathogen (Yang, 2019). *N. sichuanensis* primarily infects fishscale bamboo branches and culms through the stomatal apparatus and various wounds (Yang, 2019). Numerous pycnidia and ascostromata can be observed at infection sites, with ascostromata being particularly abundant, continuously released from November through April of the following year (Liu et al., 2022). During the disease spread period from May to October, *N. sichuanensis* mainly persists in the branches and culms of the host in the form of mycelia and conidia (Liu et al., 2022).

Plant cell walls, consisting of complex polysaccharides, which primarily include cellulose, hemicellulose, and pectin, form an effective physical barrier against fungal invasion (Underwood, 2012). Xylan, a hemicellulose characterized by a *β*-1,4-linked xylose backbone, is the predominant hemicellulose that exists in both monocots and dicots. It is integral to plant cell wall structure by providing flexibility and strength through its interactions with cellulose and lignin (Rennie and Scheller, 2014). Xylan-degrading enzymes, known as xylanases, are necessary for the full degradation of hemicellulose (Bhardwaj et al., 2019). Xylanases mainly belong to glycoside hydrolase families 10 and 11 according to their molecular weight and catalytic domains (Bhardwaj et al., 2019). GH10 xylanases generally have higher molecular weights and broader substrate specificities, whereas GH11 xylanases are smaller and exhibit better catalytic effectiveness on linear xylan chains (Liu et al., 2021).

Xylanases are crucial in the pathogenicity and infection processes of various fungal pathogens. However, reports on the contribution of GH10 family xylanases to fungal pathogenicity remain limited. For instance, the GH10 xylanase RcXYN1 from *Rhizoctonia cerealis* has been found to exacerbate disease severity during wheat infections (Lu et al., 2020). Additionally, two GH10 xylanases have been identified from the genome of *Botrytis cinerea* B05.10, but relevant experiments have not yet determined the contributions of these xylanases to the pathogenicity of this fungus (García et al., 2017). In contrast, there is considerable literature on the involvement of GH11 xylanases in fungal pathogenicity. GH11 family xylanases enable fungi to penetrate the plant cell wall by degrading xylan, which facilitates tissue colonization and nutrient acquisition for fungal growth (Wang et al., 2021). Beyond their role in cell wall degradation, GH11 family xylanases contribute to fungal development, reproduction, and virulence. For instance, in *Magnaporthe oryzae*, the rice blast fungus, the GH11 family xylanase MoXYL1A is crucial for conidiation, appressorium maturation, and virulence, underscoring its importance in effective host colonization (Shabbir et al., 2022). Furthermore, some xylanases function as protein elicitors activating PAMPs-triggered immunity (PTI) during host-pathogen interactions, triggering host defense responses that lead to cell necrosis (Enkerli et al., 1999). For example, the xylanase BcXyn11A from *Botrytis cinerea* can be recognized by plants as PAMPs and elicits plant defenses (Frías et al., 2019). This necrosis-inducing effect not only disrupts local cell integrity, providing an entry point for fungal invasion, but also suppresses nearby immune responses, creating an environment conducive to nutrient acquisition and sustained infection (Derbyshire and Raffaele, 2023). These findings emphasize that GH11 family xylanases are important components of the sophisticated strategies employed by fungal pathogens to penetrate and develop infections in their plant hosts.

Screening for xylanase genes within fungal genomes enables the systematic identification of xylanase genes potentially implicated in plant cell wall degradation, facilitating further studies through gene knockout or overexpression techniques to validate their roles in pathogenicity. Moreover, this method helps elucidate the diversity and evolutionary history of xylanase gene families, providing insights into their contribution to fungal adaptability and virulence. For example, identifying and annotating carbohydrate-active enzymes(CAZy) enhanced the comprehension of the genomic structure of plant cell wall-degrading enzymes (PCWDEs) in *Fusarium virguliforme*. Sequence and structural investigations of FvXyn11A and FvXyn11B revealed conserved residues facilitating XIP-I inhibition, with both xylanases expressed during soybean root infection (Chang et al., 2016). Comparative genomics investigations indicated that the *Verticillium dahliae* genome contains a family of six xylanases, with VdXyn4 facilitating the degradation of the plant cell wall and contributing to the pathogenicity of *V. dahliae* (Wang et al., 2021).

The genome of *Neostagonosporella sichuanensis* has been released, detailing the quantity and distribution of plant cell wall-degrading enzymes (PCWDEs) within it (Liu et al., 2024). Prior research demonstrated that *N. sichuanensis* exhibits strong xylan degradation activity, suggesting that the pathogen may employ hemicellulose degradation to penetrate plant cell walls during infection (Liu et al., 2024). Despite the well-established importance of xylanases in fungal pathogenicity, their specific role in the virulence of *N. sichuanensis* remains unknown. In this study, we comprehensively examined the involvement of GH11 family xylanases in the pathogenicity of *N. sichuanensis* in plant systems. The principal aims were to (1) determine whether these xylanases are secreted proteins and examine their location in plant cells; (2) evaluate the relationship between the enzymatic activity and cell necrosis-inducing activity; (3) determine whether *N. sichuanensis* xylanases are up-regulated during pathogen infection; and (4) investigate the contribution of xylanases to *N. sichuanensis* virulence.

## 2 Materials and methods

### 2.1 Plants, strains, and culture conditions

The wild-type strain *Neostagonosporella sichuanensis* SICAUCC 16–0001 was acquired from the Culture Collection at Sichuan Agricultural University (SICAUCC) and activated on PDA medium at 25°C for 7 days and then subcultured on fresh PDA medium at 25°C for 30 days. *Nicotiana benthamiana* was cultivated at 25°C under a 14-h light and 10-h dark cycle in an artificial climate incubator. Healthy one-year fishscale bamboo seedlings were cultivated at 25°C under a 12-h light and 12-h dark cycle in an artificial climate incubator after transplanted from Zhougongshan Town (103°2′59.87″E, 29°50′8.56″N) in Ya’an City, Sichuan Province, China, to the Chengdu Academy of Agriculture and Forestry Sciences (103°85′73.17″E, 30°70′32.61″N) in Chengdu City, Sichuan Province, China.

### 2.2 Identification and bioinformatics analysis of GH11 xylanase family proteins in *Neostagonosporella sichuanensis* SICAUCC 16–0001 genome

Xylanase family proteins were identified by querying the HMM profile of the Glyco_hydro_11 (GH11) domain (Pfam ID: PF00457) against the *Neostagonosporella sichuanensis* SICAUCC 16–0001 genome and nine other phaeosphaeriacous species (*Ampelomyces quisqualis*, *Leptosphaeria microscopica*, *Ophiobolus disseminans*, *Paraphoma chrysanthemicola*, *Parastagonospora nodorum*, *Phaeosphaeria poagena*, *Stagonospora* sp., *Setomelanomma holmii*, and *Setophoma terrestris*) using HMMER v3.4 (Potter et al., 2018) with an E-value threshold of ≤1 × 10^−5^. The returned hits with scores > 30 were selected. The genome sequences used for the xylanase proteins screening of four strains, *Leptosphaeria microscopica*, *Paraphoma chrysanthemicola*, *Phaeosphaeria poagena*, and *Setophoma terrestris* were downloaded from the JGI Genome Portal (Nordberg et al., 2014), and the genome sequences of the remaining five species were downloaded from the NCBI Genome (Sayers et al., 2022). Subsequently, these candidate proteins were searched for the Glutamic acid enzymatic activity sites and conserved glycoside hydrolase families 11 motifs using the ProSITE webserver (Sigrist et al., 2012). These sequences without enzyme active sites were discarded. The remaining sequences were submitted to SignalP v5.0 (Almagro Armenteros et al., 2019), TMHMM v2.0 (Krogh et al., 2001), BIG-PI Fungal Predictor (Eisenhaber et al., 2004), and WoLF PSORT (Horton et al., 2007) for secretory protein prediction. Protein sequences possessing a signal peptide, devoid of the transmembrane region and anchor location, and extracellular subcellular localization were identified as xylanase proteins and analyzed further. The phylogenetic tree was constructed with these selected xylanase proteins using MEGA v11 (Tamura et al., 2021) with the neighbor-joining method and visualized through Evolview v3.0 webserver (Subramanian et al., 2019). ClustalX v2.0 (Larkin et al., 2007) and ESpriPT3.0 webserver (Robert and Gouet, 2014) performed multiple sequence alignment. SOMPA (Geourjon and Deléage, 1995) and SWISS-MODEL web tools (Waterhouse et al., 2024) were used to predict the secondary and tertiary structures with the AlphaFold v2 method, respectively. The quality of the predicted models was assessed by SAVES v6.1 (Bowie et al., 1991; Lüthy et al., 1992; Colovos and Yeates, 1993; Laskowski et al., 1996) and Qmean (Benkert et al., 2011).

### 2.3 Total RNA extraction, reverse transcription, and cloning of CDS full-length of *Nsxyn1* and *Nsxyn2* genes

Fresh *Neostagonosporella sichuanensis* mycelia were collected for total RNA extraction utilizing the Quick RNA Isolation Kit (Huayueyang, Beijing, China). The concentration and purity of RNA were assessed using a NanoDrop 2000 spectrophotometer (Thermo Fisher Scientific, Waltham, MA, USA). The isolated RNA was preserved at-80°C for subsequent study. High-quality total RNA was reverse transcribed into cDNA with the PrimeScript™ RT Reagent Kit (Perfect Real Time; Takara, Beijing, China). Only two candidate xylanase genes (*Nsxyn1* and *Nsxyn2*) were successfully cloned from the cDNA of *Neostagonosporella sichuanensis* SICAUCC 16–0001. Primers (Supplementary Table S1) were designed by Primer Premier 5.0 (Premier Biosoft International, Palo Alto, CA, USA). The target fragments were amplified using 2 × TransTaq^®^ High Fidelity (HiFi) PCR SuperMix II (−dye) (TRANS, Beijing, China) and purified using a Universal DNA Purification Kit (TIANGEN, Beijing, China). Then, they were connected to pMD™-19 T vectors (Takara, Beijing, China), respectively, by the manufacturer’s guidelines. The ligation mixture was transformed into *Escherichia coli* DH5α cells, which were then spread onto antibiotic-containing Luria-Bertani (LB) agar. Positive clones were screened by colony PCR and verified by sequencing.

### 2.4 Recombinant protein expression optimization, purification, and enzyme activity assays of Nsxyn1 and Nsxyn2

*Nsxyn1* and *Nsxyn2* fragments (Amplified primers were in Supplementary Table S1), devoid of the predicted signal peptides and stop codons, were introduced into the pET-32a plasmid at the *EcoR* I and *Xho* I sites, respectively, and subsequently transformed into *Escherichia coli* BL21(DE3) for expression. To get adequate soluble proteins for subsequent analysis, expression conditions were optimized by inducing with IPTG at various concentrations and temperatures, followed by an analysis of expression levels by SDS-PAGE (Zhao et al., 2022). Large-scale protein production was carried out under optimal conditions, and the His6-tagged protein was purified utilizing a His-tag protein purification kit (Reductant&Chelator-resistant; Beyotime, Shanghai, China). Two Glu residues of the Nsxyn1rec/Nsxyn2rec mutant were replaced with Lys residues utilizing the Hieff Mut™ Site-Directed Mutagenesis Kit (Yeasen, Shanghai, China) to eliminate xylanase activity of Nsxyn1 and Nsxyn2. Following verification through sequence alignment, the Nsxyn1rec and Nsxyn2rec mutants were expressed under optimal conditions and purified as described above. Protein concentrations were then determined using a BCA protein assay kit (Beyotime, Shanghai, China).

For qualitative analysis, recombinant proteins were introduced to wells containing a medium with hardwood xylan (Beyotime, Shanghai, China) as the exclusive carbon source (Meddeb-Mouelhi et al., 2014). After 2 h of incubation, the medium was treated with 1% Congo red solution for 1 h and then eluted with 1 mol/L NaCl solution for 3 h. Hydrolytic zones were then examined using sterile eluents as negative controls. Xylanase activity was quantified by a modified DNS (dinitrosalicylic acid) assay (Fu et al., 2019) with some modifications. A mixture of 90 μL of 1% hardwood xylan solution (pH 6.5) and 10 μL of recombinant xylanase solution was incubated in PCR tubes at 45°C for 30 min. Subsequent to the reaction, 100 μL of DNS solution was immediately added, and the mixture was heated in a boiling water bath for 10 min before cooling to ambient temperature. The negative controls comprised 90 μL of 1% xylan solution (pH 6.5) and 10 μL of heat-inactivated recombinant xylanase. Absorbance was measured at 540 nm. All experiments were performed in triplicate.

### 2.5 Assessment of necrotic activity of purified Nsxyn1 and Nsxyn2 xylanase proteins in *Nicotiana benthamiana*

To assess the potential necrotic activity of the purified proteins, leaves of *Nicotiana benthamiana* were infiltrated with 10 μmol/L of each purified xylanase protein using a needleless syringe. The infiltration sites were marked, and the plants were incubated under controlled conditions at 25°C under a 14-h light and 10-h dark cycle. Leaf tissue was observed for the development of necrosis throughout 5 days post infiltration, with leaves infiltrated with buffer only used as negative controls.

### 2.6 qRT-PCR analysis

The transcript levels of *Nsxyn1* and *Nsxyn2* at different infection times in fishscale bamboo were quantified by qRT-PCR on a CFX96™ real-time system (Bio-Rad, Hercules, CA, USA). Specific primers for amplification were designed according to the coding regions of *Nsxyn1* and *Nsxyn2,* with the *Neostagonosporella sichuanensis* elongation factor 1 alpha gene as an internal control (the coding sequence is detailed in Supplementary Table S2). The qRT-PCR reaction setup and conditions followed those described by Liu et al. (2019). A no-template control (ddH_2_O) was included. The infection timepoint exhibiting the lowest expression served as the baseline (assigned a value of 1.0). Relative expression levels were calculated utilizing the 2^-∆∆Ct^ method (Ritter and Schulz, 2004; Zhu et al., 2015). Each qRT-PCR experiment was performed in triplicate. Data were analyzed and plotted using GraphPad version 8.4.2, with statistical significance assessed by unpaired Student’s *t*-test.

### 2.7 Signal peptides secretion function detection of Nsxyn1 and Nsxyn2

To detect whether the signaling peptides of Nsxyn1 and Nsxyn2 proteins possess secretory functions, the relevant signaling peptide sequences were inserted into the pSUC2 vector and transformed into *Saccharomyces cerevisiae* YTK12 competent cells, which are lacking invertase secretion. Transformants were selected on CMD-W medium and cultured at 30°C for 48 h. Positive colonies were transferred to the YPRAA medium to evaluate the secretory activity and incubated at 30°C for 48 h. The CMD-W and YPRAA mediums were prepared according to Huang’s method (Huang C. P. et al., 2023). The secretory function was further confirmed by staining with 2,3,5-triphenyl tetrazolium chloride (TTC), as described by Yin et al. (2018). The appearance of a red precipitate indicated successful secretion of the expressed proteins.

### 2.8 Subcellular localization assays and transient expression in *Nicotiana benthamiana* of Nsxyn1 and Nsxyn2

To investigate the subcellular localization of Nsxyn1 and Nsxyn2 proteins in planta and assess their necrosis-inducing activity, the coding sequences of *Nsxyn1* and *Nsxyn2* (without stop codons) were ligated to the 5′ end of the enhanced green fluorescent protein (EGFP) gene, driven by the MAS promoter (Amplified primers were in Supplementary Table S1). These constructs were then inserted into the pCAMBIAsuper1300-EGFP vector at the *Kpn*I and *Xba*I restriction sites to generate recombinant expression vectors, pCAMBIAsuper1300:*Nsxyn1*-EGFP and pCAMBIAsuper1300:*Nsxyn2*-EGFP.

Onion epidermal cells and *Arabidopsis thaliana* protoplasts were used for subcellular localization analysis. Disinfected onion inner epidermal slices (~1 cm^2^) were incubated in *Agrobacterium* suspensions (GV3101) containing either *Nsxyn1*-EGFP, *Nsxyn2*-EGFP, or the empty pCAMBIAsuper1300-EGFP vector as a control, for 30 min. The epidermal slices were then placed on MS medium and incubated at 25°C under a 14-h light and 10-h dark cycle. Fluorescence was observed 24 h later using an Ultra-High Resolution Confocal Microscope (Leica, Wetzlar, Germany), with excitation at 488 nm. Moreover, protoplasts were prepared from the leaves of 3–4 week-old *A. thaliana* seedlings for protoplast localization. The *Nsxyn1* and *Nsxyn2* plasmids were co-transformed with an SV40 NLS plasmid into the protoplasts via PEG4000-mediated transformation. After overnight incubation at 23°C under low light conditions, the protoplasts were centrifuged at 400 rpm for 5 min, and the supernatant was discarded. Fluorescent signals were visualized using a confocal laser microscope (FV1000). Fluorescence detection parameters were GFP excitation at 488 nm, mCherry excitation at 561 nm, and chloroplast autofluorescence at 640 nm.

For necrosis induction assays, *Agrobacterium tumefaciens* GV3101 (pSoup-19) harboring the *Nsxyn1* or *Nsxyn2* constructs was infiltrated into the leaves of *Nicotiana benthamiana*. Empty vector pCAMBIAsuper1300-EGFP-MGS served as a negative control. After 3 days of incubation at 25°C with a 14-h light and 10-h dark cycle, the infiltrated leaves were collected, and the development of necrosis was assessed.

### 2.9 Generation of gene deletion mutants and complementation

*Nsxyn1* and *Nsxyn2* mutants were created via targeted gene disruption using homologous recombination. Gene-specific left, and right flanking regions (1,000–1,500 bp) along with the hygromycin resistance flanking region were amplified and inserted into the pCE-Zero vector (detailed primers are in Supplementary Table S1). Using the method described by Liang et al. (2024), the knockout construct was introduced into *Neostagonosporella sichuanensis* SICAUCC 16–0001 via protoplast transformation. Successful mutant generation was verified by PCR analysis.

For the complementation of mutants, the full-length *Nsxyn1* and *Nsxyn2* genes, including their native promoter regions, were inserted into the pEASY-NeoR vector, which contains a neomycin resistance (NeoR) marker (detailed primers are in Supplementary Table S1). These constructs were reintroduced into the respective mutant strains. Complemented strains were selected on G418-containing mediums, and successful integration was verified by PCR.

### 2.10 Phenotypic and virulence analysis of *Nsxyn1* and *Nsxyn2* gene deletion mutants and complemented strains

To assess the impact of *Nsxyn1* and *Nsxyn2* deletions on fungal development and stress adaptation, a comprehensive phenotypic analysis was conducted on the gene deletion mutants, complemented strains, and wild-type controls. Growth rates were determined by measuring colony diameters on a PDA medium incubated at 25°C over a 30-day incubation period. Sporulation was quantified by counting conidia produced on the PDA plates after 30 days using a hemocytometer. Spore morphology was examined under a light microscope. At the same time, conidial germination rates were assessed by incubating conidia in sterile distilled water for 24 h at 25°C, followed by counting germinated spores under the microscope.

Oxidative and osmotic stress assays were performed to evaluate stress tolerance. Strains were grown on a PDA medium supplemented with 10 μmol/L Congo red for oxidative stress and either 1 mol/L NaCl or 0.02% SDS for osmotic stress. Colony growth was monitored by measuring the diameters after a 30-day incubation period. To evaluate the capacity of these strains to degrade primary cell wall components, they were cultured on the PDA mediums containing 1% (w/v) cellulose, xylan, or pectin as the sole carbon source. Enzyme activity was quantified by measuring the diameter of clear zones after Congo red staining, providing insight into the ability to degrade cell wall polysaccharides of these strains.

For virulence assessment, *Nsxyn1* and *Nsxyn2* gene deletion mutants, complemented strains, and the wild-type strains were cultured on PDA at 25°C for 30 days. Single conidia were isolated and transferred to fresh PDA medium for 15-day incubation. Conidia were harvested and diluted to a concentration of 1 × 10^6^ conidia/mL in sterile distilled water. Surface-sterilized host plant leaves were wounded with a sterile needle, and 10 μL of the conidial suspension was applied to each wound. Inoculated plants grow at 25°C with 80% relative humidity. Disease symptoms were monitored daily. Lesion sizes were recorded at 30 days post-inoculation to determine virulence. The assays were repeated in triplicate in each treatment.

Data analysis was performed using One-way ANOVA followed by Duncan’s multiple range test in SPSS version 27.0 (SPSS Inc., Shanghai, China). Graphical data visualization was conducted using GraphPad Prism 8.4.2 to illustrate significant differences in disease indices among strains.

## 3 Results

### 3.1 Identification of GH11 domain-containing xylanases in *Neostagonosporella sichuanensis*

**Figure 1:**
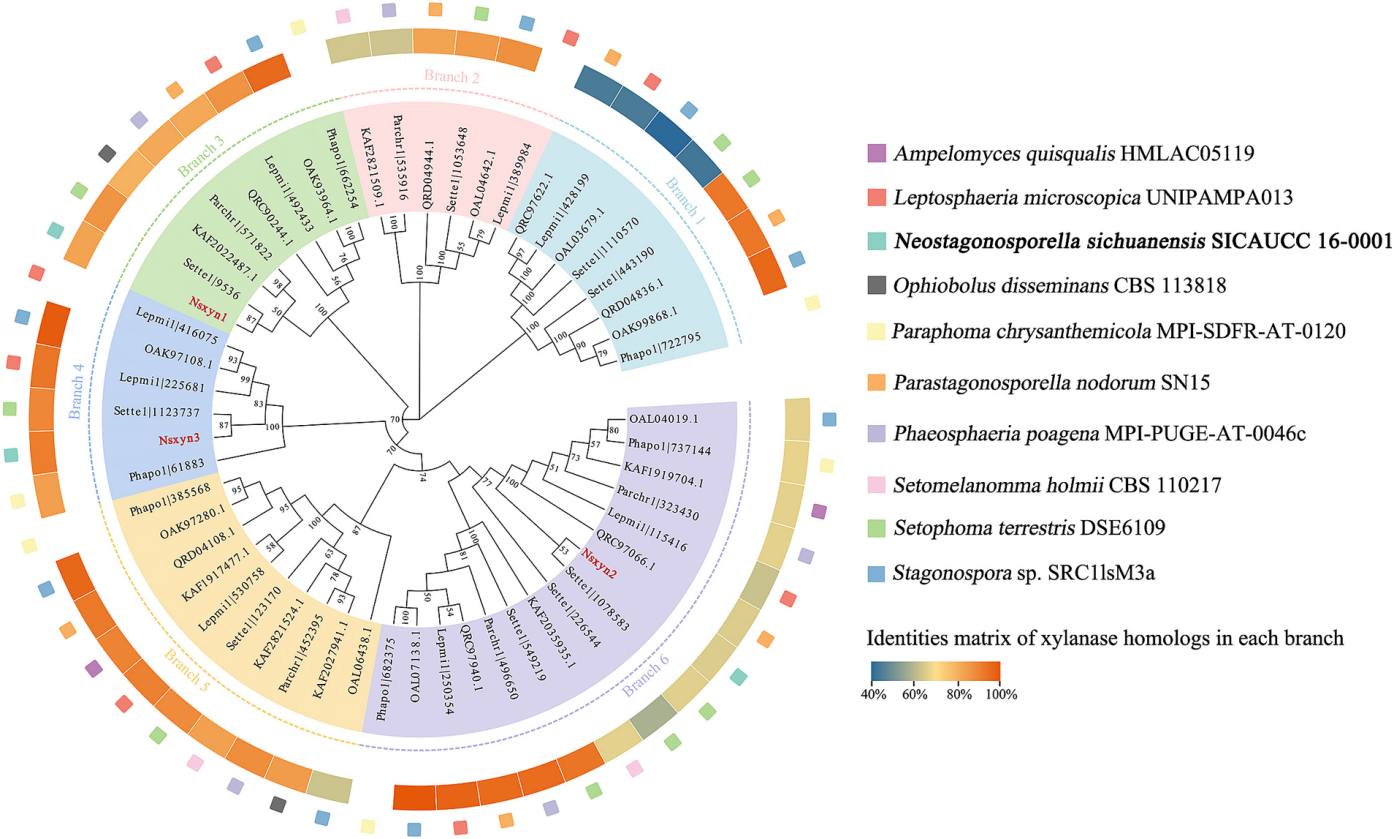
Phylogenetic analysis of GH11 xylanase proteins between *Neostagonosporella sichuanensis* and nine other phaeosphaeriacous species constructed using the neighbor-joining method. We select the first sequence in each branch in a counterclockwise direction for sequence similarity comparison with other sequences within the branch. Nsxyn1, Nsxyn2, and Nsxyn3 are highlighted in bold red. In the phylogenetic tree, nodes with support values below 50% were not displayed.

**Figure 2:**
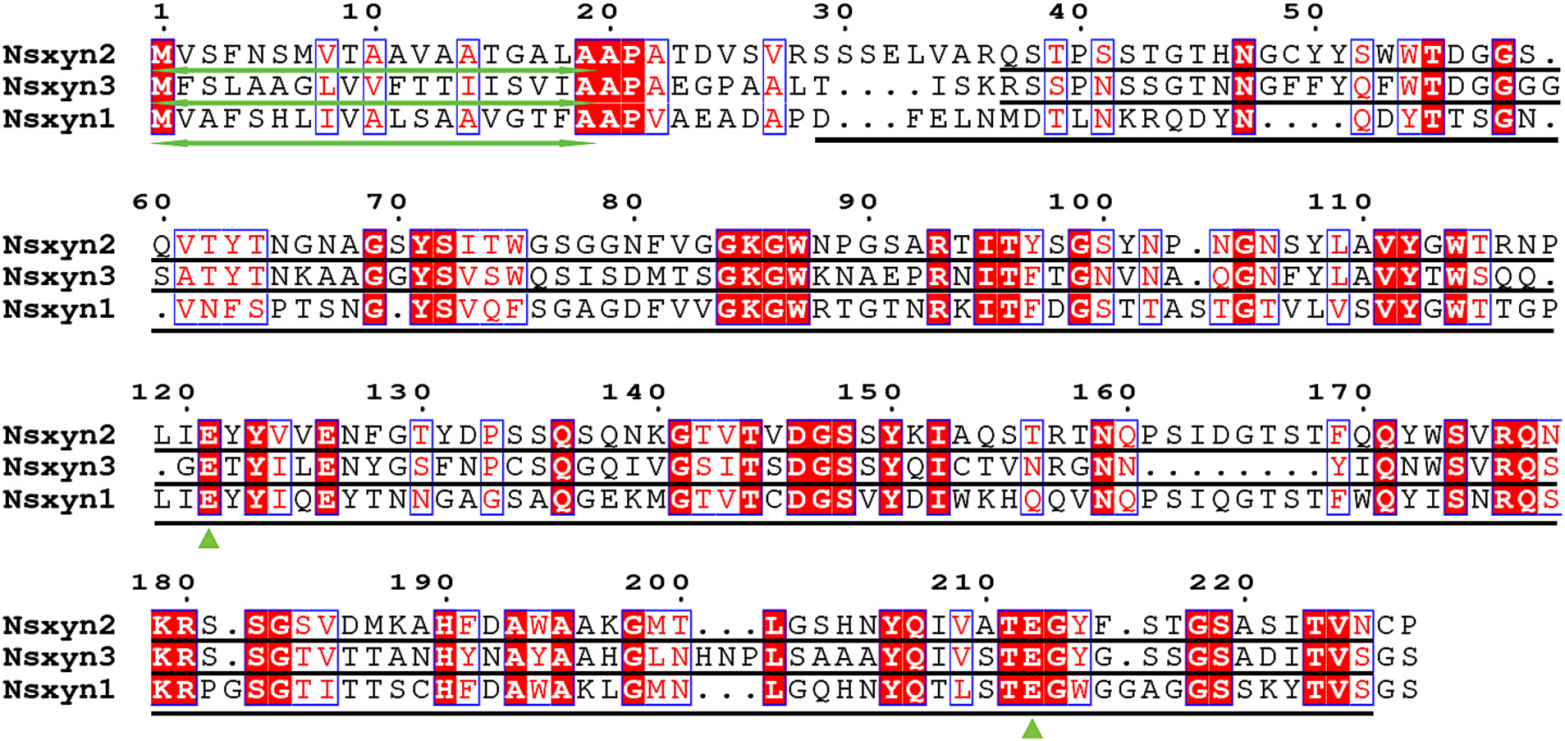
Sequence alignments were performed for Nsxyn1, Nsxyn2 and Nsxyn3. Numbers denote the positions of the amino acid residues. Bidirectional green arrows mark signal peptides, while black lines represent GH11 domains. Green triangles highlight the two conserved glutamate enzymatic activity sites. The alignment sequences of each site are highlighted with red characters on a white background with blue frames when the amino acid similarity in the same column is greater than 0.7 and highlighted with white characters on a red background with blue frames when they are strictly conserved in the column. Amino acids with a similarity of less than 0.7 in the same column are highlighted with black characters on a white background.

Three xylanases containing GH11 domain proteins (Nsxyn1-Nsxyn3) were identified in the genome of *Neostagonosporella sichuanensis* SICAUCC 16–0001 through bioinformatics analysis. These xylanases were identified as secreted proteins with signaling peptides, lacking transmembrane regions and GPI-anchor signals (Supplementary Table S3). The same screening method predicted 51 xylanase proteins from nine other phaeosphaeriacous species (Supplementary Table S3). These xylanase proteins and Nsxyn1-Nsxyn3 were subjected to phylogenetic analysis with the neighbor-joining method. The generated phylogenetic tree clustered the proteins into six distinct clades, designated Branch1 to Branch6, widely represented among the analyzed phaeosphaeriacous species. Most xylanases exhibited relatively higher sequence similarity within the same branches, indicating a degree of conservation within the Phaeosphaeriaceae family (Figure 1). However, sequence alignment analysis revealed low homology between the GH11 domains of the three xylanases from *N. sichuanensis*, with pairwise identities of 44.04% between Nsxyn1 and Nsxyn2, 29.44% between Nsxyn1 and Nsxyn3, and 47.67% between Nsxyn2 and Nsxyn3 (Figure 2). Despite the low sequence homology, all three xylanases shared two conserved catalytic sites associated with glutamic acid residues (Figure 2).

### 3.2 Structural model prediction and quality assessment of Nsxyn1-Nsxyn3 proteins of *Neostagonosporella sichuanensis*

**Figure 3:**
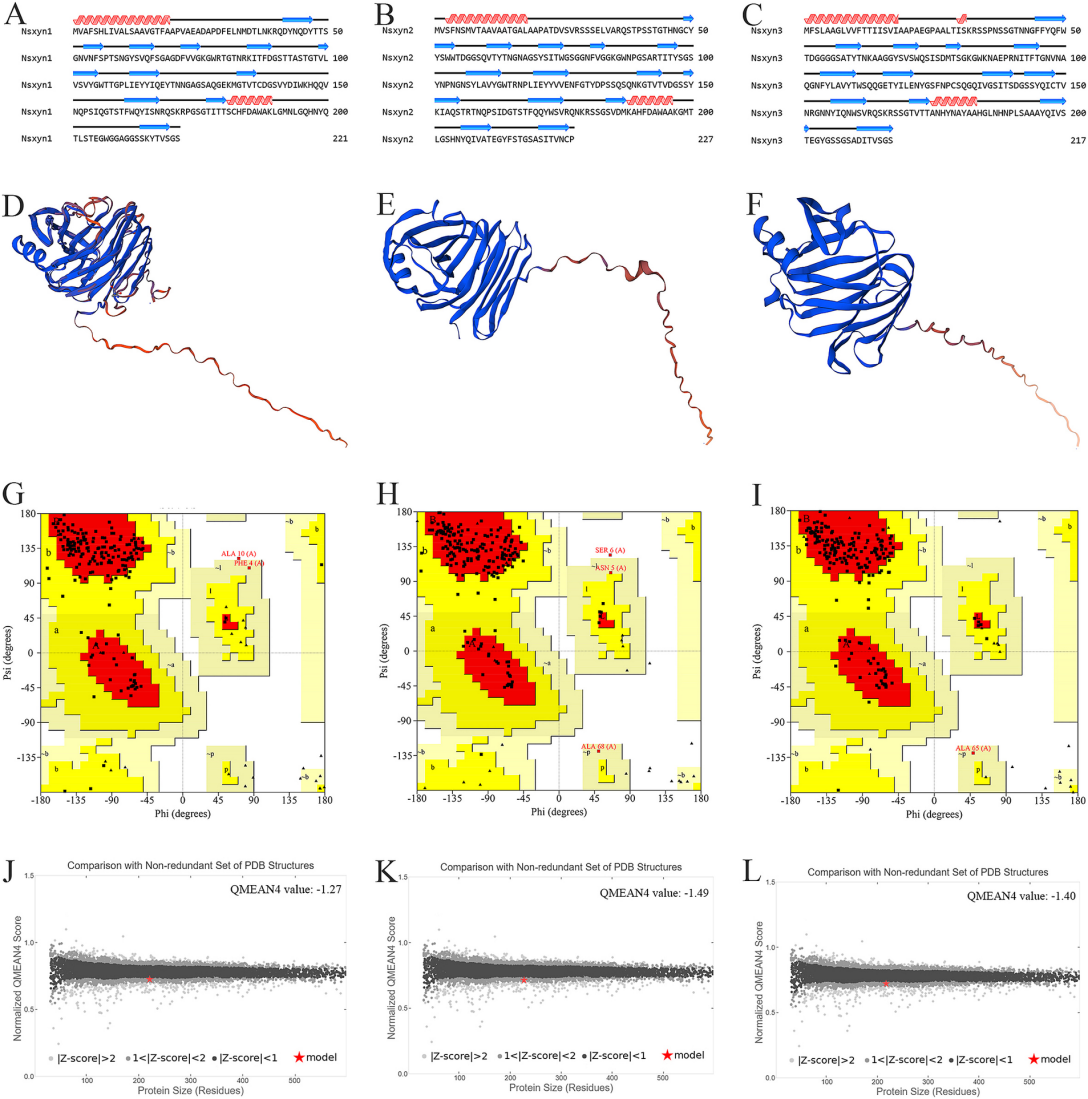
Structural analysis and model quality assessment of Nsxyn1, Nsxyn2 and Nsxyn3. **(A–C)** Secondary structures. The red helices, blue arrows, and black lines indicate alpha helices, extended strands, and random coils. (**D–F**) 3D structures with “confidence (gradient)” color schemes of Nsxyn1, Nsxyn2, and Nsxyn3 were constructed from AlphaFold DB models of 4ZRR9_LEPMJ and W6YRM9_COCMI, respectively. The bluer the models are, the higher the confidence level, while the redder the models are, the lower the confidence level. The coverage and sequence identity of Nsxyn1 **(D)** were 100 and 82.35%, respectively. The coverage and sequence identity of Nsxyn2 **(E)** were 100 and 87.22%, respectively. The coverage and sequence identity of Nsxyn3 **(F)** were 99 and 84.19%, respectively. **(G–I)** Ramachandran plots of the protein conformations generated by PROCHECK. There are 88.6% core region, 10.3% allow region, 0.5% general allow region, and 0.5% disall region of all amino acid residues of Nsxyn1 **(G)**, respectively, while 88.0% core region, 10.4% allow region, 1.0% general allow region, and 0.5% disall region of all amino acid residues of Nsxyn2 **(H)**. In comparison, 90.7% core region, 8.8% allow region, 0.5% general allow region, and 0.0% disall region of the amino acid residues of Nsxyn3 **(I)**. **(J–L)** Comparison with the non-redundant set of PDB structures using QMEAN.

The number and locations of alpha helices and extended strands were similar between Nsxyn1, Nsxyn2, and Nsxyn3 proteins according to the analysis of their secondary structures (Figures 3A–C) and tertiary structures (Figures 3D–F), leading us to hypothesize that all three proteins have similar functions. The tertiary structure quality of the three xylanase proteins was assessed using the SAVES pipeline and Qmean. The overall quality factor of the tertiary structure of Nsxyn1, Nsxyn2, and Nsxyn3 was 90.45, 89.11, and 88.89, respectively, as determined by the ERRAT program and Nsxyn1 and Nsxyn3 passed the quality test of the Verify3D program, in contrast to Nsxyn2 where less than 80% (77.97%) of the residues had an averaged 3D-1D score > = 0.1. Nevertheless, the Ramachandran plots generated by PROCHECK showed that over 90% of the amino acid residues of all three proteins were in acceptable regions (Figures 3G–I). On the other hand, the Nsxyn1, Nsxyn2, and Nsxyn3 models evaluated by Qmean had Z-scores of-1.27, −1.49, and-1.40, respectively (Figures 3J–L). The SAVES pipeline and the Qmean evaluation results revealed that the tertiary structures of all three protein models predicted by the SWISS-MODEL using the Alphafold v2 method were consistently of good quality and suitable for further experimental study.

### 3.3 Cloning two candidate xylanases from *Neostagonosporella sichuanensis*

Two candidate xylanase genes (*Nsxyn1* and *Nsxyn2*) were cloned from the cDNA of *Neostagonosporella sichuanensis* SICAUCC 16–0001 (Supplementary Figure S1); however, *Nsxyn3* was not obtained despite numerous attempts. The sequences of two genes obtained by cloning, *Nsxyn1* and *Nsxyn2*, were identical to the sequences from the genome annotation of *Neostagonosporella sichuanensis* SICAUCC 16–0001, reflecting the accuracy of the genome assembly and annotation results. The functions of *Nsxyn1* and *Nsxyn2* genes were then further investigated.

### 3.4 Optimization of expression conditions and purification of Nsxyn1, Nsxyn2, Nsxyn1^SM113–205^, and Nsxyn2^SM121–212^ recombinant proteins

Recombinant plasmids pET-32a-*Nsxyn1* and pET-32a-*Nsxyn2* were successfully constructed and transformed into *Escherichia coli* BL21 (DE3) competent cells (Supplementary Figure S2). Expression parameters, including IPTG concentration, induction duration, and temperature, were carefully optimized to improve Nsxyn1 and Nsxyn2 protein yield and enhance protein solubility. The findings indicated that IPTG concentration did not impact Nsxyn1 and Nsxyn2 protein expression, so the lowest IPTG concentration of this experiment, 0.2 mM, was chosen to continue optimizing other expression conditions (Supplementary Figures S3A,B). Similarly, the expression of Nsxyn1 was not affected by the duration of induction, so 2 h was optimal (Supplementary Figure S3C). Conversely, 5 h was chosen for pET-Nsxyn2 induction due to the significantly increased amount of induced protein compared to the previous hours (Supplementary Figure S3D). The expression of Nsxyn1 and Nsxyn2 proteins was highest at 30°C (Supplementary Figures S3E,F). The optimal screening conditions induced the expression of Nsxyn1 and Nsxyn2 proteins before purification.

To determine whether the two Glu sites in Nsxyn1 and Nsxyn2 proteins are essential for the enzyme activity, we conducted site-directed mutagenesis genes, Nsxyn1^SM113–205^ and Nsxyn2^SM121–212^. Subsequently, they were transferred to *E. coli* BL21 (DE3) for overexpression using the previously optimized conditions.

Solubility detection experiments showed that Nsxyn1, Nsxyn2, Nsxyn1^SM113–205^, and Nsxyn2^SM121–212^ were present as soluble proteins (Supplementary Figures S3G,H). The Nsxyn1, Nsxyn2, Nsxyn1^SM113–205^, and Nsxyn2^SM121–212^ proteins were purified before detection of enzyme activity.

The Nsxyn1 and Nsxyn2 recombinant proteins have xylanase activity and can induce the necrosis phenotype of *Nicotiana benthamiana* independently of xylanase activity.

**Figure 4:**
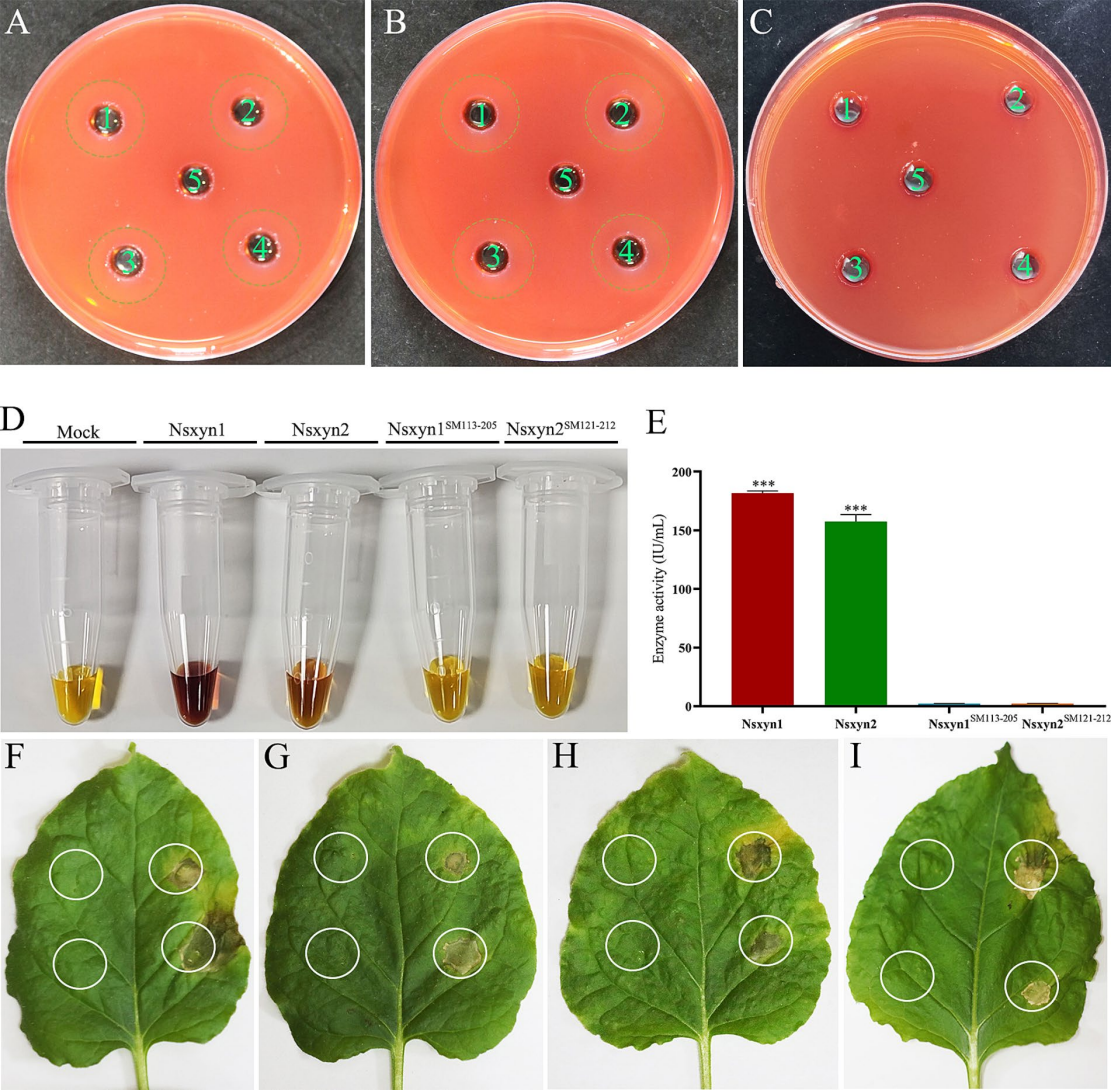
Qualitative and quantitative determination of the enzyme activity of purified recombinant Nsxyn1 and Nsxyn2 proteins. Determination of the ability of purified Nsxyn1 **(A)** and Nsxyn2 **(B)** proteins to degrade xylan, respectively. 1–4: Recombinant Nsxyn1/Nsxyn2 protein. 5: Control eluent. The hydrolysis circle reflects the ability of Nsxyn1/Nsxyn2 to degrade xylan, and the larger the hydrolysis circle, the greater the ability of the recombinant Nsxyn1/Nsxyn2 protein to degrade xylan. **(C)** Determination of the xylanase activity of purified Nsxyn1^SM113–205^ and Nsxyn2^SM121–212^ proteins to degrade xylan. 1–2: Nsxyn1^SM113–205^ recombinant protein. 3–4: Nsxyn2^SM121–212^ recombinant protein. 5: Eluent as control. **(D,E)** Determination of xylanase activity of Nsxyn1/Nsxyn2 proteins and two site-directed mutants. Xylan was used as the only carbon source for induction in each case. Error bars indicate the standard errors (SE) of the mean. Asterisks *** indicates statistical significance at *p* < 0.001 based on unpaired Student’s t-tests. Nsxyn1 **(F)**, Nsxyn2 **(G)**, Nsxyn1^SM113–205^ **(H)**, and Nsxyn2^SM121–212^ **(I)** inducing cell necrosis in *Nicotiana benthamiana* leaves. The white ring on the left side of the leaf indicates the blank control for the eluent, while the white circle on the right indicates the location of the purified proteins.

The purified Nsxyn1 and Nsxyn2 proteins exhibited the ability to hydrolyze xylan to form hydrolytic rings when they were added to the small holes of the medium with xylan as the sole carbon source (Figures 4A,B). On the other hand, Nsxyn1^SM113–205^ and Nsxyn2^SM121–212^ lost the ability to degrade xylan (Figure 4C). The above findings align with the quantitative enzyme activity analysis of the purified protein using xylan as substrate (Figures 4D,E).

Injection of purified Nsxyn1 and Nsxyn2 proteins into *Nicotiana benthamiana* leaves resulted in necrosis approximately 5 days after treatment (Figures 4F,G). To determine whether this necrosis was related to the enzymatic activity of these proteins, site-directed mutagenesis was used to generate catalytic site mutants (Nsxyn1^SM113–205^ and Nsxyn2^SM121–212^) in both proteins. Despite the loss of enzymatic activity, the mutant proteins still induced necrosis in *N. benthamiana* leaves, comparable to the unmutated proteins (Figures 4H,I). These findings suggested that the necrosis-inducing activity of these xylanase proteins is independent of their enzymatic function.

### 3.5 Quantitative analysis of *Nsxyn1* and *Nsxyn2* expression

**Figure 5:**
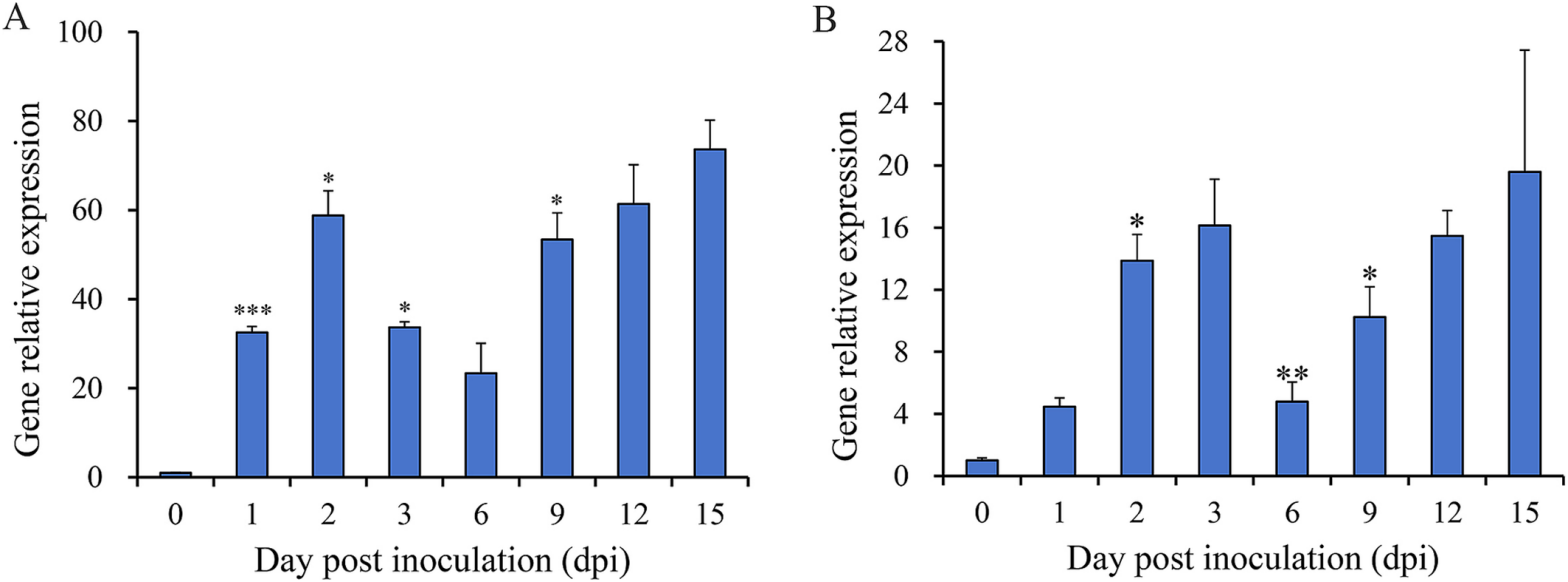
Relative expression levels of *Nsxyn1* **(A)** and *Nsxyn2* **(B)** at different infection times of *Neostagonosporella sichuanensis*. Error bars represent standard errors (SE). Asterisks *, **, and *** on the SE line are statistically significant differences at *p* < 0.05, *p* < 0.01, and *p* < 0.001, respectively, as determined by unpaired Student’s *t*-tests.

To assess the involvement of *Nsxyn1* and *Nsxyn2* in the infection process of *Neostagonosporella sichuanensis*, their expression levels at different periods of infection with fishscale bamboo were determined using qRT-PCR (Figure 5). Under non-inductive conditions, the expression levels of both genes were at their lowest, normalized to 1. Expression analysis revealed significant up-regulation of *Nsxyn1* and *Nsxyn2* during the infection process, with *Nsxyn1* showing a particularly strong increase, reaching an 80-fold up-regulation at 15 days post-inoculation. These results suggest that *Nsxyn1* and *Nsxyn2* are strongly induced during infection.

### 3.6 Signal peptides of Nsxyn1 and Nsxyn2 proteins have secretory functions

**Figure 6:**
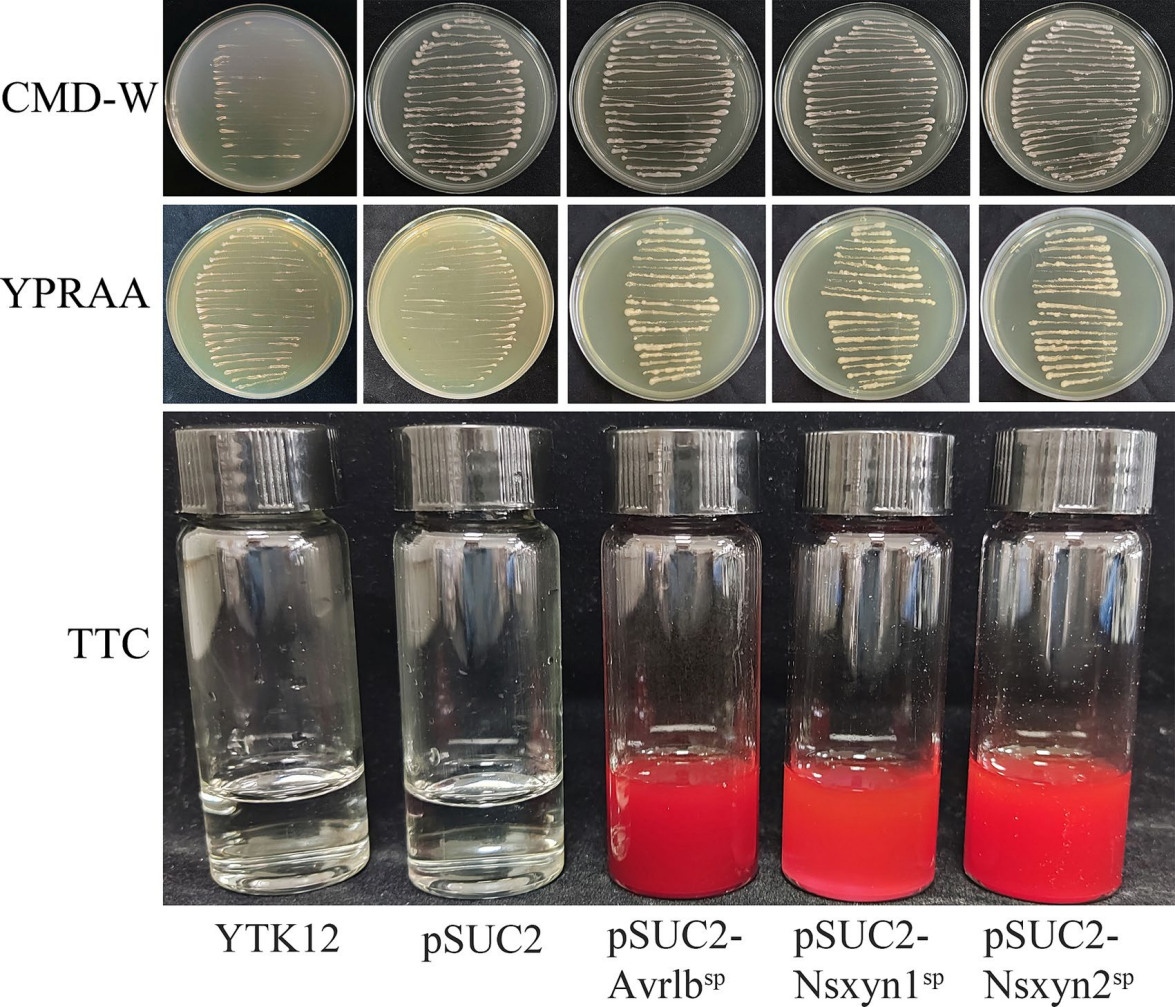
Functional assessment of the Nsxyn1 and Nsxyn2 signal peptides. YTK12 strains carrying pSUC2-Nsxyn1^SP^, pSUC2-Nsxyn2^SP^ together with pSUC2-Avr1b^SP^ (positive control) are capable of growth on CMD-W medium and YPRAA media, whereas the YTK12 strain devoid of vector and possessing pSUC2 empty vector (negative controls) do not. Additionally, the YTK12 strains containing pSUC2-Nsxyn1^SP^, pSUC2-Nsxyn2^SP^, and the positive control change the color of the reaction mixture from colorless to bright and dark red in the TTC assay, whereas the negative controls show no color change.

The *Nsxyn1* and *Nsxyn2* genes were inserted into the pSUC2 vector and subsequently transformed into the YTK12 yeast strain. Both transformants grew on CMD-W and YPRAA selective media, indicating successful gene expression (Figure 6). Similarly, the positive control strain harboring the pSUC2-Avr1b^SP^ construct also exhibited normal growth, whereas the negative control showed no growth, confirming the validity of the experimental system. Further TTC staining analysis revealed that YTK12 strains expressing Nsxyn1 and Nsxyn2 produced a red precipitate. These results strongly suggest that the proteins encoded by these genes contain functional signal peptides that facilitate secretion.

### 3.7 Subcellular localization of Nsxyn1 and Nsxyn2 proteins and their role in inducing necrosis in *Nicotiana benthamiana*

**Figure 7:**
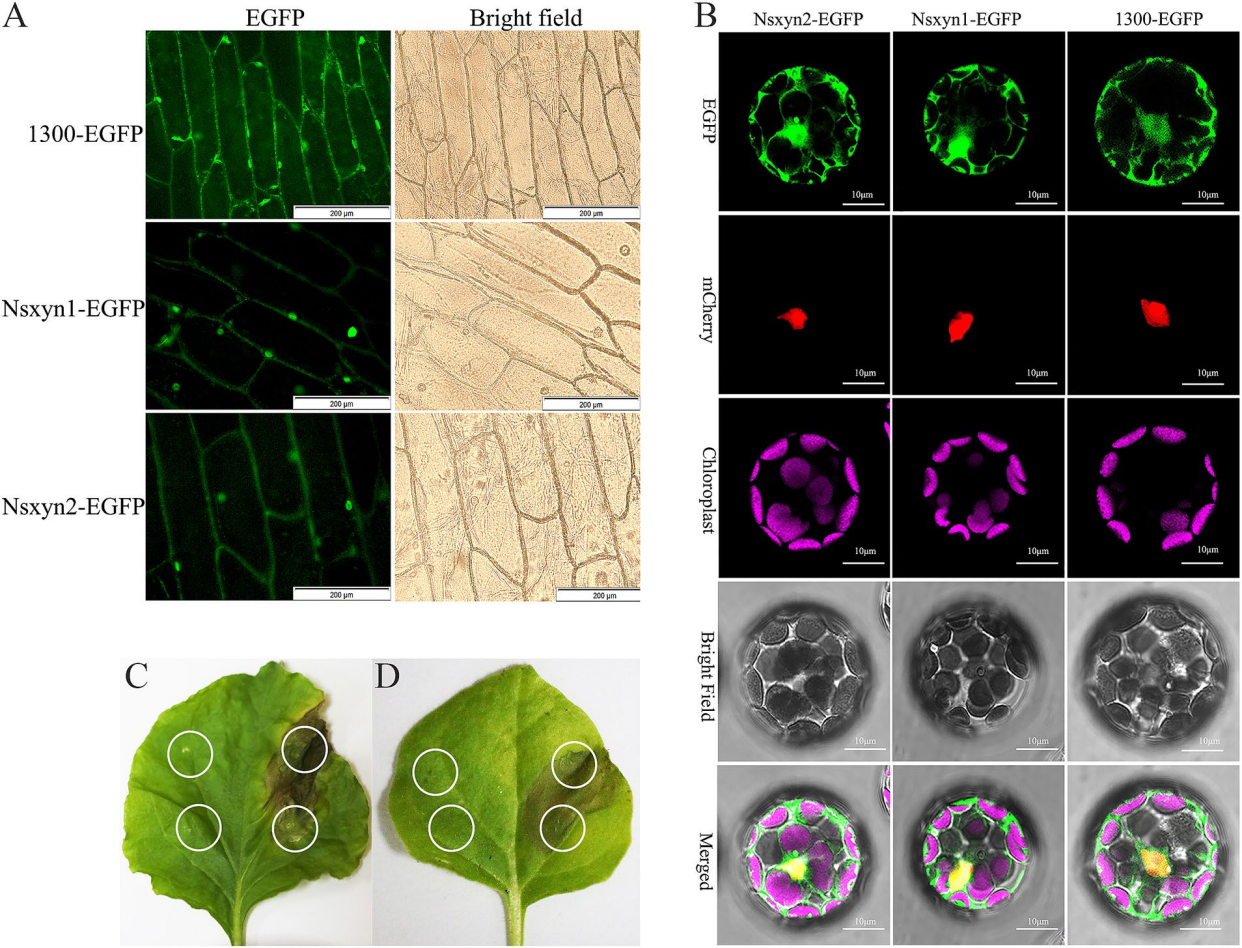
Subcellular localization of the Nsxyn1 and Nsxyn2 proteins is examined in onion epidermal cells **(A)** and *Arabidopsis thaliana* protoplasts **(B)**. The pCAMBIA1300-EGFP vector is used as a control. In *Arabidopsis thaliana* protoplasts, red fluorescence indicates nuclear markers, while purple fluorescence is due to chloroplast autofluorescence, and the green fluorescence emitted by the pCAMBIA1300-GFP vector is visible. Membrane and nuclear markers exhibit red fluorescence. Transient expression of *Nsxyn1* **(C)** and *Nsxyn2* **(D)** are both observed to induce necrosis in *N. benthamiana* leaves. The white ring on the left side of the leaf denotes the negative control, where only EGFP-expressing *Agrobacterium* suspensions are infiltrated. The white circles on the right indicate the regions infiltrated with *Nsxyn1*-EGFP **(C)** and *Nsxyn2*-EGFP **(D)** *Agrobacterium* suspensions, respectively.

The plasmid maps and electrophoretic analysis during vector construction are presented in Supplementary Figure S4. All fluorescence signals were effectively recorded using confocal laser scanning microscopy in the onion epidermal cells (Figure 7A) and *Arabidopsis thaliana* protoplasts (Figure 7B) expressing the Nsxyn1 and Nsxyn2. Results from the transient expression system showed that onion epidermal cells with empty PCAMBIAsuper1300-GFP carriers emitted green fluorescence throughout the cell structure. However, EGFP green fluorescence was mainly distributed in the nuclear onion cells with pCAMBIAsuper1300-*Nsxyn1*-EGFP or pCAMBIAsuper1300-*Nsxyn2*-EGFP overexpression carriers. Furthermore, when examined in *A. thaliana* protoplasts, the Nsxyn1 and Nsxyn2 xylanases displayed a dual localization pattern in both the nucleus and the cytoplasm. Together, Nsxyn1 and Nsxyn2 proteins were localized in the nucleus and cytoplasm.

Transient expression of *Nsxyn1*-EGFP and *Nsxyn2*-EGFP in *Nicotiana benthamiana* leaves resulted in localized cell necrosis, as evidenced by visible necrotic lesions at the infiltration sites (Figures 7C,D). In contrast, the EGFP control did not induce any signs of necrosis, and the leaves remained healthy. This suggested that Nsxyn1 and Nsxyn2 possessed necrosis-inducing activity in plant cells, independent of the fluorescent tag.

### 3.8 Obtaining gene knockout and knockout complementation

Deletion and complementation strains for both genes were generated to investigate the role of *Nsxyn1* and *Nsxyn2* in cell wall degradation (Supplementary Figures S5, S6). It was observed that *Neostagonosporella sichuanensis* exhibited minimal growth on plates containing either 90 μg/mL hygromycin B or 200 μg/mL G418 (Supplementary Figure S7), leading to the selection of these concentrations for transformant screening. The deletion strains of *Nsxyn1* and *Nsxyn2* were verified by PCR analysis (Supplementary Table S1), and positive transformants were chosen for further study (Supplementary Figures S8, S9).

### 3.9 Nsxyn1 and Nsxyn2 proteins display cell wall-degradation activity

**Figure 8:**
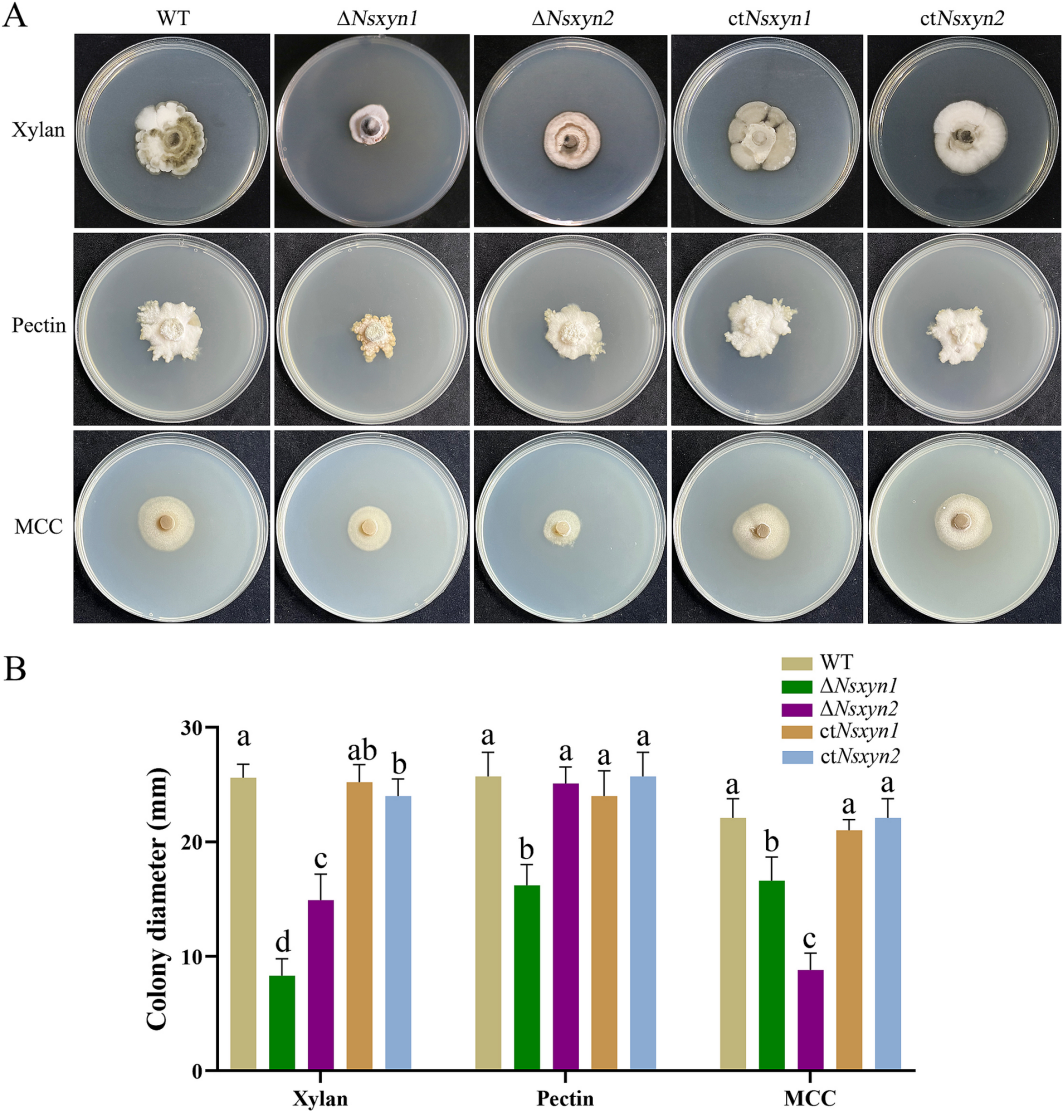
Growth observations of the *Nsxyn1* and *Nsxyn2* knockout mutants and complement strains on basic C’zapek medium, utilizing xylan, pectin, or microcrystalline cellulose (MCC) as the sole carbon source. **(A)** Photographs of the growth phenotypes were taken after incubation at 25°C for 30 days. **(B)** Comparison of colony diameters. Error bars represent the standard errors of the mean. Distinct lowercase letters indicate significant differences in colony diameter among the various strains on the same type of culture media at *p* < 0.05, as determined by one-way ANOVA.

The capacity of the deletion mutants (Δ*Nsxyn1* and Δ*Nsxyn2*) and the complementation strains (ct*Nsxyn1* and ct*Nsxyn2*) to degrade cell wall components was evaluated on the C’zapek media that was devoid of carbon sources but supplied with xylan, cellulose, or pectin. On the C’zapek medium with xylan as the sole carbon source, colony diameters of Δ*Nsxyn1* and Δ*Nsxyn2* mutants were significantly smaller than those of the wild type (Figure 8). Additionally, the colony diameter of the Δ*Nsxyn1* mutants was unexpectedly decreased on C’zapek media with cellulose or pectin, while the Δ*Nsxyn2* mutants showed a similar reduction only when grown on C’zapek medium with pectin. The complementation strains, ct*Nsxyn1*, and ct*Nsxyn2*, completely restored cellulose and pectin degradation capacity and partially restored the potential for xylan degradation.

### 3.10 Analysis of phenotypic, sporulation, and spore germination of transformation

**Figure 9:**
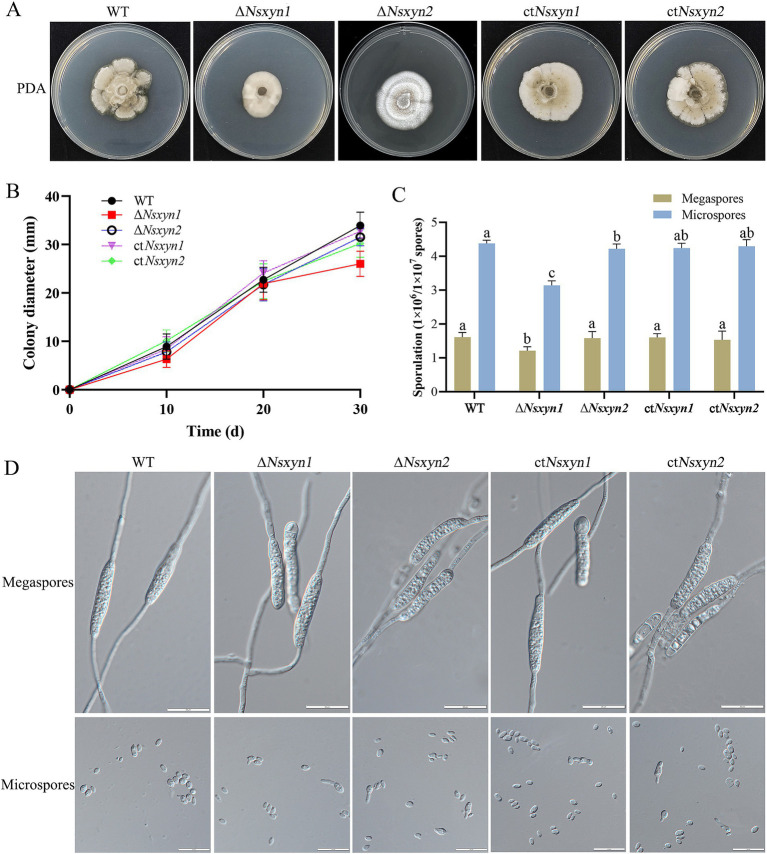
Colony phenotypes **(A)**, mycelial growth rates **(B)**, conidial sporulation statistics **(C)**, and germination morphology of megaspores and microspores **(D)** from different strains (wild-type strain, *Nsxyn1* and *Nsxyn2* deletion strains, and their corresponding complementation strains) grown under light/dark (12 h/12 h) conditions at 25°C for 30 days. Error bars represent the standard error of the mean. Different lowercase letters indicate significant differences in megaspore/microspore sporulation among the various strains at *p* < 0.05, as determined by one-way ANOVA. The unit for counting megaspores is 1 × 10^6^ spores, while the unit for counting microspores is 1 × 10^7^ spores.

To examine the impact of *Nsxyn1* and *Nsxyn2* gene deletion on the growth of *Neostagonosporella sichuanesis*, mycelial plugs of wild type, Δ*Nsxyn1* and Δ*Nsxyn2* mutants, and the ct*Nsxyn1* and ct*Nsxyn2* complement strains were inoculated on PDA medium under light/dark (12 h/12 h) conditions at 25°C for 30 days. A significant decrease in colony diameter and spore production was observed in the Δ*Nsxyn1* mutant relative to the wild type (Figures 9A–C). In contrast, no strong negative effect on the growth rate was observed in the Δ*Nsxyn2* mutant (Figures 9A,B). However, it was found that the colony morphology of the Δ*Nsxyn2* mutant changed, shifting from a smooth surface to a cotton-like appearance, with a corresponding reduction in microconidia production compared to the wild type (Figures 9A,C). Spore production only statistically partially recovered, and the growth rate was restored after complementation. Additionally, the conidial morphology of all transformant strains did not differ significantly from that of the wild type, and all strains were able to germinate normally (Figure 9D).

### 3.11 Stress response of transformants

**Figure 10:**
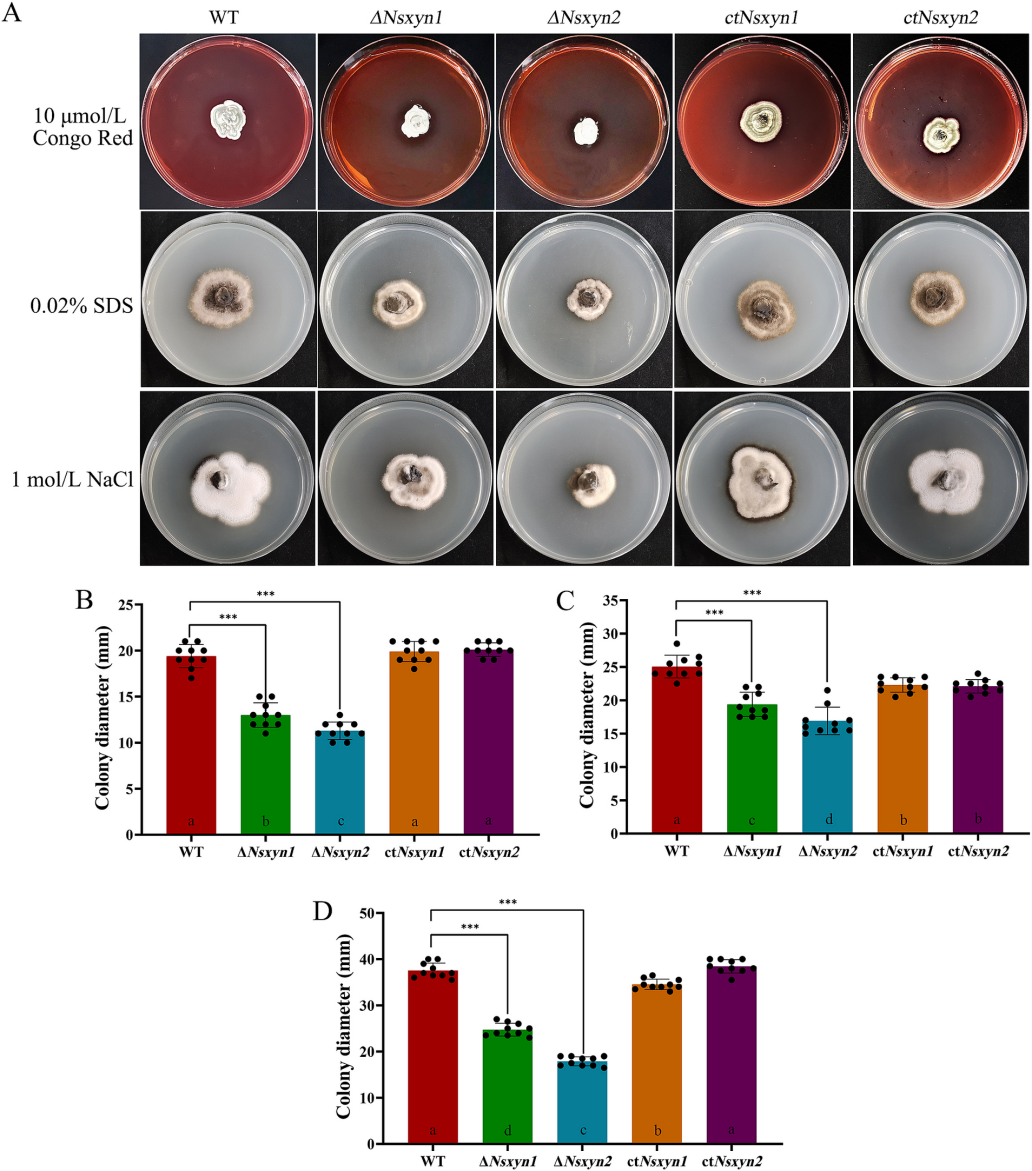
Sensitivity of *Nsxyn1* and *Nsxyn2* mutants to cell wall stress-inducing agents. Colony morphology **(A)** and colony diameter measurements **(B–D)** of the wild-type strain (WT), *Nsxyn1* and *Nsxyn2* deletion mutants (Δ*Nsxyn1* and Δ*Nsxyn2*), and their corresponding complement strains (ct*Nsxyn1* and ct*Nsxyn2*) were assessed after culturing on PDA media supplemented with various cell wall perturbing agents for 30 days. **(B)** 10 μmol/L Congo Red (CR); **(C)** 0.02% sodium dodecyl sulfate (SDS); **(D)** 1 mol/L NaCl. Colony diameters were averaged from ten technical replicates, and error bars represent standard errors. Asterisks (***) indicate a significant difference (*p* < 0.001) based on unpaired Student’s t-tests. Different letters on the bar charts denote statistically significant differences at *p* < 0.05 (one-way ANOVA).

The integrity of the fungal cell wall is crucial for successful host cell infection, as it maintains the shape of the fungal cell and regulates interactions with the external environment (Cabib et al., 2001). Continuous cell wall remodeling is required for proper fungal growth and development (Bowman and Free, 2006). We assessed the effects of cell wall-disrupting agents on the growth of the Δ*Nsxyn1* and Δ*Nsxyn2* mutant strains (Figure 10). Colony size measurements were employed to quantify the growth inhibition rate, revealing that the Δ*Nsxyn1* and Δ*Nsxyn2* strains exhibited significant sensitivity to cell wall stressors, including Congo red, SDS, and NaCl. Notably, the Δ*Nsxyn2* strain displayed greater sensitivity than the Δ*Nsxyn1* strain.

### 3.12 Pathogenicity test of transformants

**Figure 11:**
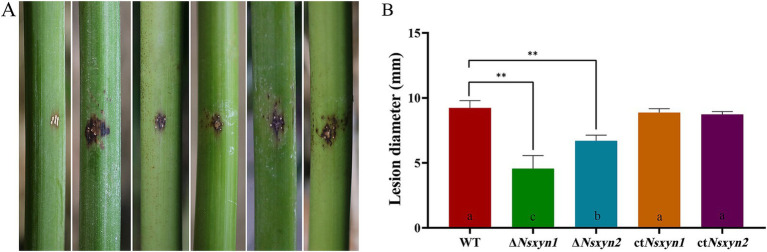
Photographs depicting the symptoms of infection on fishscale bamboo culms **(A)** and the corresponding statistics of lesion size **(B)** after 30 days of inoculation with conidial suspension. From left to right, the treatments include sterile water, conidial suspensions of wild-type, and the *Nsxyn1* and *Nsxyn2* detection mutants (Δ*Nsxyn1* and Δ*Nsxyn2*), along with their corresponding complementary strains (ct*Nsxyn1* and ct*Nsxyn2*). Wounds were inoculated with sterile water as a control. Each treatment was conducted in triplicate. Different lowercase letters indicate statistically significant differences among the treatments under the same stress conditions, as determined by a one-way ANOVA at *p* < 0.05. Asterisks (**) indicate a significant difference (*p* < 0.01) based on unpaired Student’s t-tests.

Pathogenicity assays were conducted to determine the roles of Nsxyn1 and Nsxyn2 in rhombic-spot disease on living fishscale bamboo culms. The wild-type strain caused typical necrotic symptoms. In contrast, while the Δ*Nsxyn1* and Δ*Nsxyn2* mutants displayed similar symptoms, their virulence was significantly reduced (Figure 11). Complementation strains ct*Nsxyn1* and ct*Nsxyn2* restored most of the virulence phenotype. These findings underscore the critical roles of Nsxyn1 and Nsxyn2 in the pathogenicity of *N. sichuanensis*.

## 4 Discussion

Bamboos are widely used in construction, furniture, and ecological restoration (Ndavaro et al., 2022). However, bamboo culm diseases have significantly challenged bamboo production and quality. Despite the increasing prevalence of reported bamboo culm diseases (Huang L. et al., 2023; Sonali et al., 2023; Yang et al., 2024), the pathogenic mechanisms of bamboo culm pathogens, including *Neostagonosporella sichuanensis* (the primary pathogen of rhombic-spot disease), remain few investigated. Xylanases have been identified as key virulence factors in numerous previous studies (Nguyen et al., 2011; Yu et al., 2016; Yu et al., 2018; Wang et al., 2021). Our early experiments have demonstrated that *N. sichuanensis* exhibited good xylan degradation capabilities (Liu et al., 2024), but what role xylanases play in the rhombic-spot disease remains uninvestigated. The high-quality whole genome sequences of *N. sichuanensis* SICAUCC 16–0001 (GenBank accession number: JAUGWR000000000) have been published in the NCBI database, which provides a valuable resource for gene identification and functional validation. Here, we identified the xylanases belonging to the GH11 family in the *N. sichuanensis* SICAUCC 16–0001 genome, in which two xylanases (Nsxyn1 and Nsxyn2) were deeply studied for their roles in pathogenesis. Despite numerous attempts, we could not obtain the *Nsxyn3* gene; therefore, synthesizing the *Nsxyn3* gene fragment for future functional studies would be a promising approach. We identified Nsxyn1 and Nsxyn2 as secretory proteins possessing conserved functional domains and enzyme activity sites through multiple sequence alignments. The secretion potential and enzyme activity of Nsxyn1 and Nsxyn2 proteins were verified through signal peptide secretion assays and enzyme activity detection, suggesting their potential roles in host cell wall degradation. Expression analysis via qRT-PCR showed that *Nsxyn1* and *Nsxyn2* were up-regulated to varying degrees during the infection of fishscale bamboo, with *Nsxyn1* showing the most significant upregulation. The upregulation of xylanase genes has been demonstrated to be a critical factor in the pathogenicity of fungi, particularly during interaction with host plants (Brito et al., 2006; Lai and Liou, 2018). The significant expression levels of *Nsxyn1* and *Nsxyn2* further indicate that they play a vital role in degrading the cell wall of fishscale bamboos and potentially function as effectors that modulate plant immune responses. Moreover, the significant decrease in pathogenicity observed in the *Nsxyn1* and *Nsxyn2* mutants provides convincing evidence of its functional involvement. However, xylanases are not always essential for pathogenicity, as demonstrated with other fungi, including *Cochliobolus carbonum*, *Fusarium oxysporum* f. sp. *Lycopersici,* and *Fusarium graminearum* (Apel, 1993; Gómez-Gómez et al., 2002; Sella et al., 2013).

Accumulating evidence suggests that plant cell wall-degrading enzymes (PCWDEs), including xylanases, can induce host defense responses and promote cell death independently of their enzymatic activity (Enkerli et al., 1999; Noda et al., 2010; Gui et al., 2017). For example, the strong cell death and PAMP-triggered immunity (PTI) induced by *BcXyl1* were observed in several plants and independent of its enzyme activity (Yang et al., 2018). These PCWDEs were defined as a class of necrosis-inducing proteins (NIPs). Although Nsxyn1 and Nsxyn2 are xylanase proteins, they can cause cell necrosis in *Nicotiana benthamiana* independently of xylanase activity, indicating that a specific motif or protein domain mediates cell death-inducing activities (Zhu et al., 2017). The PCWDEs inducing cell necrosis have been reported to act as pathogen-associated molecular patterns (PAMP) and possibly recognized by plant leucine-rich repeat receptor-like proteins (LRR-RLPs) (Sabnam et al., 2023). PAMP usually triggers early plant immune responses, such as oxidation bursts, MAPK phosphorylation, ROS production, and callose accumulation (Nicaise et al., 2009; Peng et al., 2018). Furthermore, the RLP-SOBIR1-BAK1 complex is essential for the downstream signaling and cell death activity of PCWDEs (Frías et al., 2019; Wang et al., 2022). The potential of Nsxyn1 and Nsxyn2 to induce plant immune responses, particularly in fishscale bamboo, and whether the RLP-SOBIR1-BAK1 complex mediates their cell death-inducing activity requires further investigation. The mechanism by which plant cells receive the xylanase elicitor remains unclear. Previous studies suggest that xylanase recognition may be directly detected by plant cells through a receptor for this protein or indirectly through fragments of plant cell walls generated by its enzymatic activity (Bucheli et al., 1990; Hanania and Avni, 1997). Our study observed that the xylanases Nsxyn1 and Nsxyn2 localized to cytoplasm and nuclei in *Arabidopsis thaliana* protoplasts. Localization within the cytoplasm may enable these xylanases to interact directly with host cellular components, thereby modulating plant defense responses. For example, the cytoplasmic effector Avr1b from *Phytophthora sojae* has been shown to interfere with host immune signaling pathways by directly interacting with host proteins, ultimately promoting disease development (Dou et al., 2008). The dual localization of Nsxyn1 and Nsxyn2 suggests their involvement in multiple stages of the infection process, with nuclear localization possibly indicating a role in influencing host transcriptional responses (Kim et al., 2020; Harris et al., 2023). A 25-residue peptide originating from xylanase BcXyn11A was reported to induce PTI immune responses, including cell necrosis, ROS burst, and seedling growth inhibition (Frías et al., 2019). Notably, despite the low similarity of Nsxyn1 (43.53% identity) and Nsxyn2 (59.39% identity) to BcXyn11A, both proteins contained two similar conserved regions with four consecutive amino acid residues to BcXyn11A, namely YGWT and YYIV (with YYIQ in Nsxyn1 and YYVV in Nsxyn2). These observations indicate that Nsxyn1, Nsxyn2, and BcXyn11A potably share functional commonalities. Moreover, the virulence role of BcXyn11A is linked to its necrotizing activity (Frías et al., 2019); further investigation is required to determine whether Nsxyn1 and Nsxyn2 contribute to virulence through a similar mechanism.

The growth rate or sporulation rate of many xylanase mutants of fungi decreased significantly (Yu et al., 2016; Shabbir et al., 2022; Cui et al., 2024). The deletion of *Nsxyn1* had a notable adverse impact on fungal growth rates and sporulation quantity, whereas only a reduction in microconidial production was observed in *Nsxyn2* mutants. These results suggest that *Nsxyn1* may play a key role in fungal growth and nutrient acquisition, whereas other genes may supplement the function of *Nsxyn2*. To substantiate this hypothesis, the effect of both deletions on fungal growth can be further investigated through a double knockout experiment. Conidia are critical for the pathogenicity of *Neostagonosporella sichuanensis* as they serve as important primary inocula for infecting stomatas or wounds of fishscale bamboo through wind or rain drops (Liu et al., 2022). The severity of rhombic-spot disease correlates directly with the conidia count within rhombic-spot lesions. The number of conidia in the *Nsxyn1* mutant decreased significantly, suggesting its importance in asexual sporulation.

Xylan has a highly complex structure and is a significant component of the hemicellulose found in plant cell walls, whose complete degradation requires the coordinated activity of multiple enzymes (Sarangi and Tahatoi, 2024). In this study, the knockdown of *Nsxyn1* and *Nsxyn2*, respectively, significantly reduced the ability of *Neostagonosporella sichuanensis* to degrade xylanase, especially *Nsxyn1*, but did not wholly prevent colony growth. There are multiple xylanase family genes in the *N. sichuanensis* genome, and other xylanase genes may partially or wholly compensate for the function of the knocked-down genes, helping to maintain a certain degree of xylan degradation and colony growth. In addition, the knockdown of *Nsxyn1* and *Nsyxn2* not only made a difference in the ability of *N. sichuanensis* to degrade xylan but also affected pectin and cellulose degradation. One possibility is that changes in xylanase production affect the overall secretion system or metabolic pathways of the microorganism, indirectly influencing the production of other enzymes like pectinase. This is supported by studies showing that enzyme secretion and growth are tightly linked in lignocellulose-degrading microorganisms, and altering one enzyme may impact others (Thite and Nerurkar, 2018).

The polysaccharide network in fungal cell walls is crucial for regulating the flow of chemical substances between the fungal cells and their external environment (Díaz-Jiménez et al., 2012; Garcia-Rubio et al., 2020). The heightened sensitivity of *Nsxyn1* and *Nsxyn2* mutants to cell wall-disrupting osmolytes implies that these genes are crucial for maintaining cell wall integrity.

## 5 Conclusion

In summary, this study identified and cloned two xylanase genes, *Nsxyn1* and *Nsxyn2*, and demonstrated that the corresponding proteins are secretory proteins localized in the nucleus and cytoplasm of *Arabidopsis thaliana*. Both proteins exhibit significant xylanase enzyme activity but also induce cell necrosis in *Nicotiana benthamiana* independently of this activity. Further research is necessary to clarify the exact regions within these enzymes recognized by plants as pathogen-associated molecular patterns (PAMPs). Additionally, our results suggest that *Nsxyn1* and *Nsxyn2* are essential for xylan degradation, adaptation to osmotic and oxidative stress, and full pathogenic virulence. Deletion of *Nsxyn1* notably slowed fungal growth and reduced spore production, whereas only a reduction in microconidial production was observed in *Nsxyn2* mutants. These findings lead us to hypothesize that *Nsxyn1* plays a key role in fungal growth and nutrient acquisition, whereas the function of *Nsxyn2* may be compensated by other genes. Exploring the effects of double knockout mutants of these xylanase genes will be essential to elucidate their functional redundancy and cooperative roles in pathogenicity. These findings will enhance our understanding of the mechanisms by which xylanases contribute to the pathogenicity of fungi.

## Data Availability

The datasets presented in this study can be found in online repositories. The names of the repository/repositories and accession number(s) can be found in the article/Supplementary material.
