# Supplementary material for: Global characterization of GH11 family xylanases genes in Neostagonosporella sichuanensis and functional analysis of Nsxyn1 and Nsxyn2
Source: Front Microbiol. 2024 Nov 21;15:1507998. doi: 10.3389/fmicb.2024.1507998 (PMC11618621; doi:10.3389/fmicb.2024.1507998)
Supplement: Supplementary file 1 [file Data_Sheet_1.pdf]

Supplementary Material

1 Supplementary Figures

1.1 Supplementary Figure 1

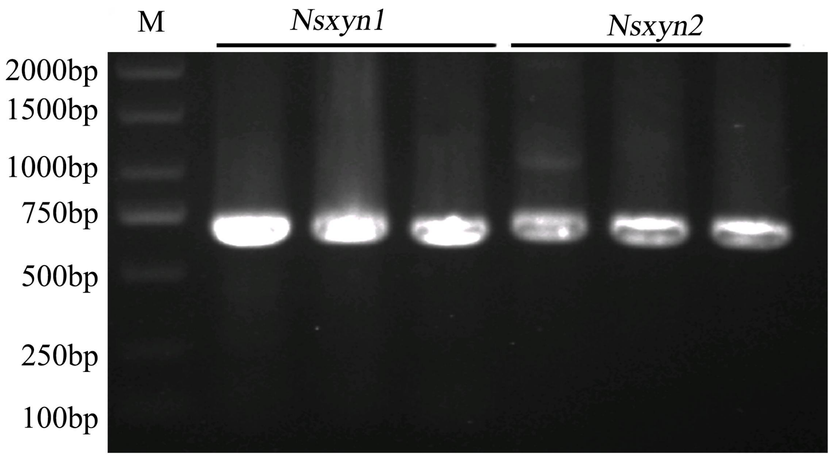

Supplementary Figure 1. Electrophoretogram of CDS fragments of target genes. M: DL2000 DNA marker.

1.2 Supplementary Figure 2

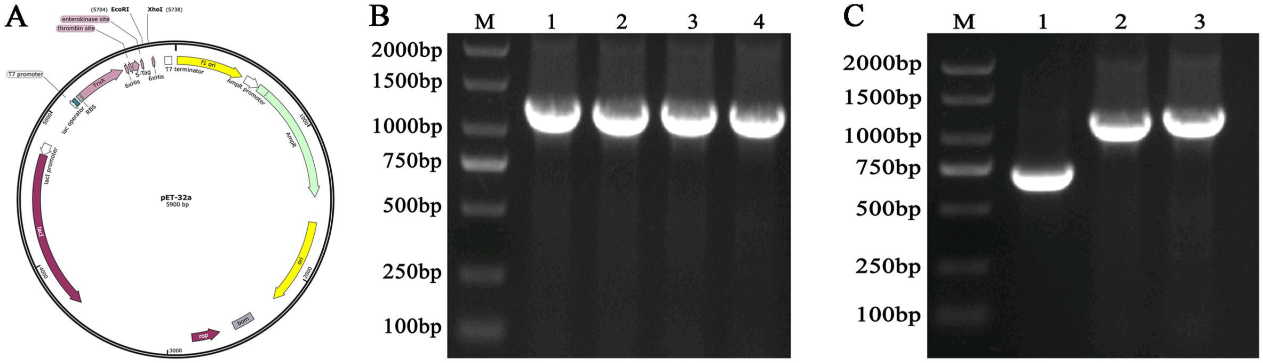

Supplementary Figure 2. Plasmid map of pET-32a and vector construction and plasmid transformation identification results. (**A**) Plasmid map of pET-32a. (**B**) M: DL2000 DNA marker; lanes 1-4: Electrophoretogram of recombinant plasmid pET-*Nsxyn1* transformed strains. (**C**) M: DL2000 DNA marker; lanes 1-4: Electrophoretogram of recombinant plasmid pET-*Nsxyn2* transformed strains.

1.3 Supplementary Figure 3

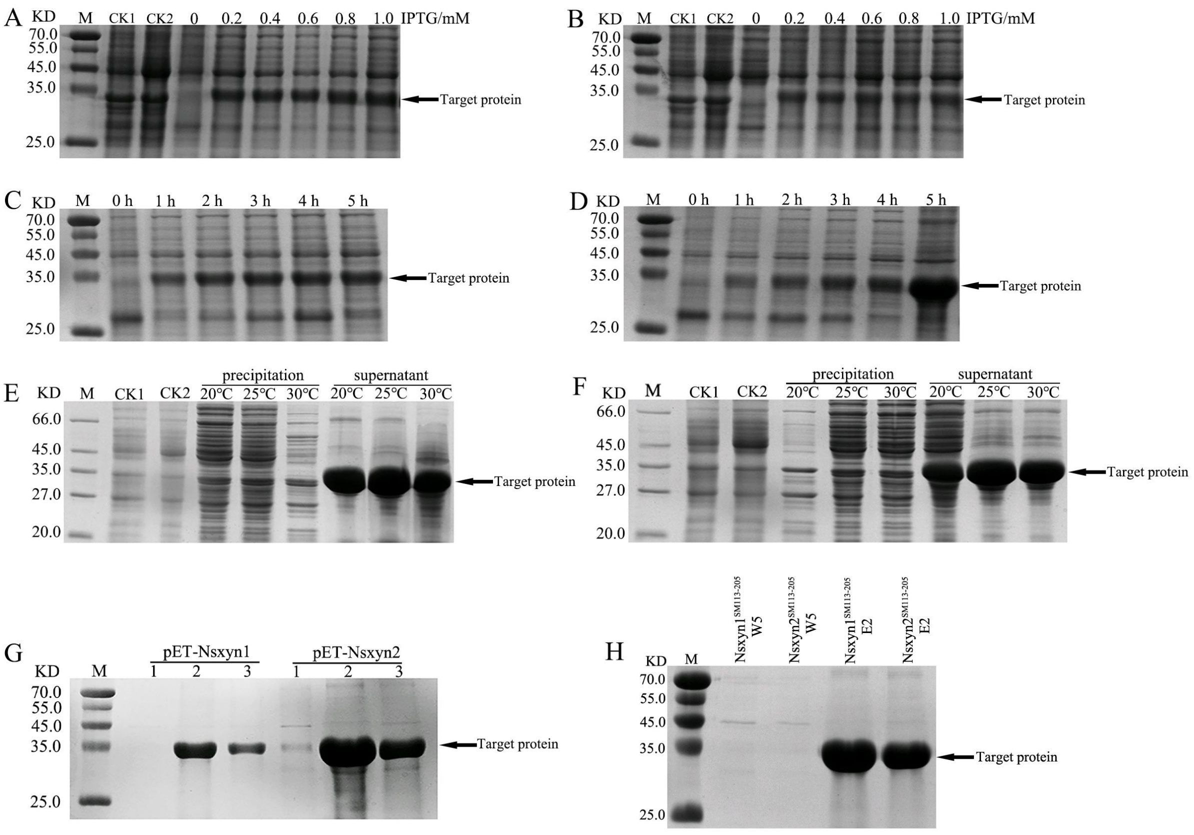

Supplementary Figure 3. The SDS-PAGE analysis of recombinant Nsxyn1 and Nsxyn2 protein. (**A-B**) The expression of recombinant Nsxyn1 and Nsxyn2 proteins was induced using IPTG concentrations of 0, 0.2, 0.4, 0.6, 0.8, and 1.0 mM. (**C-D**) The time-dependent expression profiles of recombinant Nsxyn1 and Nsxyn2 protein were analyzed at intervals of 0, 1, 2, 3, 4, and 5 hours. (**E-F**) Protein solubility and induction efficiency were assessed at 20 ° C, 25 ° C, and 30 ° C for each recombinant protein. (**G**) Purification of Nsxyn1 and Nsxyn2 proteins. 1: Electrophoretic detection of wash supernatant after fifth washing during the purification process of Nsxyn1/Nsxyn2 proteins; 2-3: Purified proteins of Nsxyn1/Nsxyn2 proteins. (**H**) Purification of Nsxyn1$^{SM113-205}$ and Nsxyn2$^{SM113-205}$ proteins. lanes 1 and 3: Electrophoretic detection of wash supernatant after fifth washing during the purification process of Nsxyn1$^{SM113-205}$/Nsxyn2$^{SM113-205}$ proteins; lanes 2 and 4: Purified proteins of Nsxyn1$^{SM113-205}$/Nsxyn2$^{SM113-205}$ proteins. M indicates the protein marker. CK1 is the pET-32a vector control expressed in *Escherichia coli* BL21 (DE3) without IPTG induction at 25℃ in 3 hours, and CK2 represents the pET-32a vector induced with 0.6 mM IPTG under the same conditions.

1.4 Supplementary Figure 4

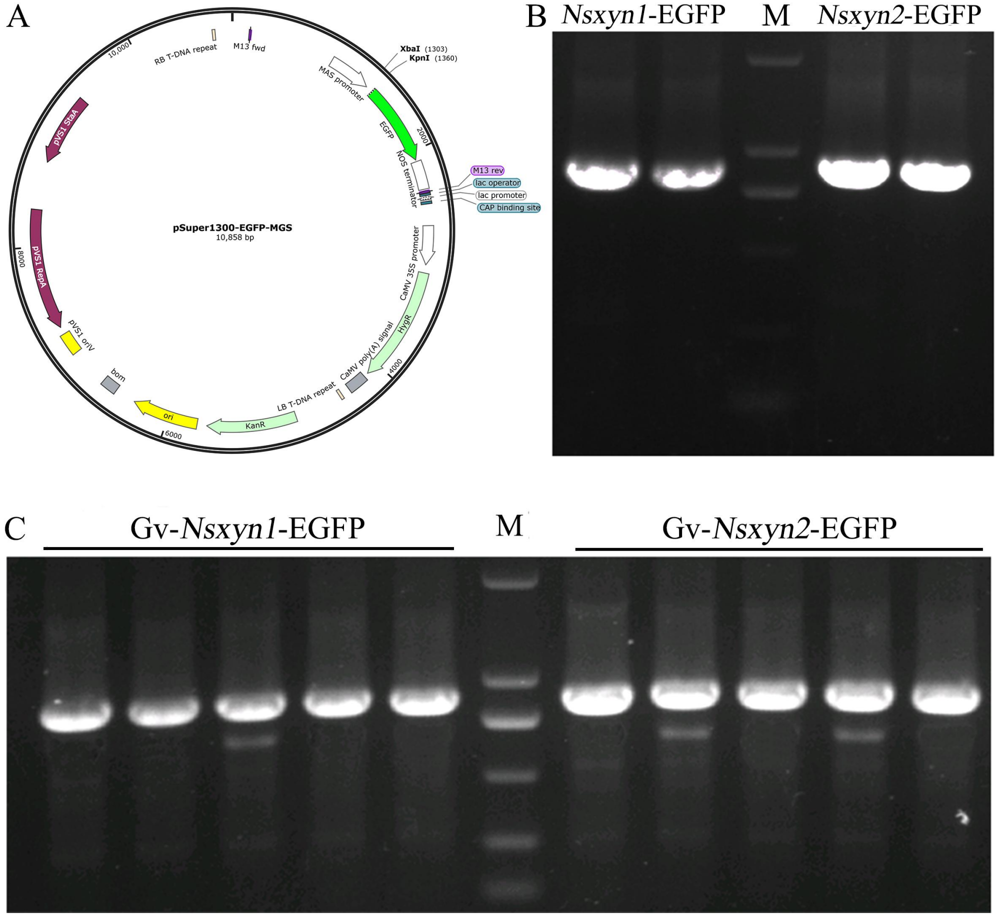

Supplementary Figure 4. Plasmid map of pCAMBIAsuper1300-EGFP-MCS the identification results of plasmid transformation. (**A**) Plasmid map of pCAMBIAsuper1300-EGFP-MCS. (**B**) *Nsxyn1*-EGFP and *Nsxyn2*-EGFP were introduced into trans1-T1 competent cells. M: DL2000 DNA marker. (**C**) *Nsxyn1*-EGFP and *Nsxyn2*-EGFP were introduced into *Agrobacterium* GV3101 (pSoup) competent cells. M: DL2000 DNA marker.

1.5 Supplementary Figure 5

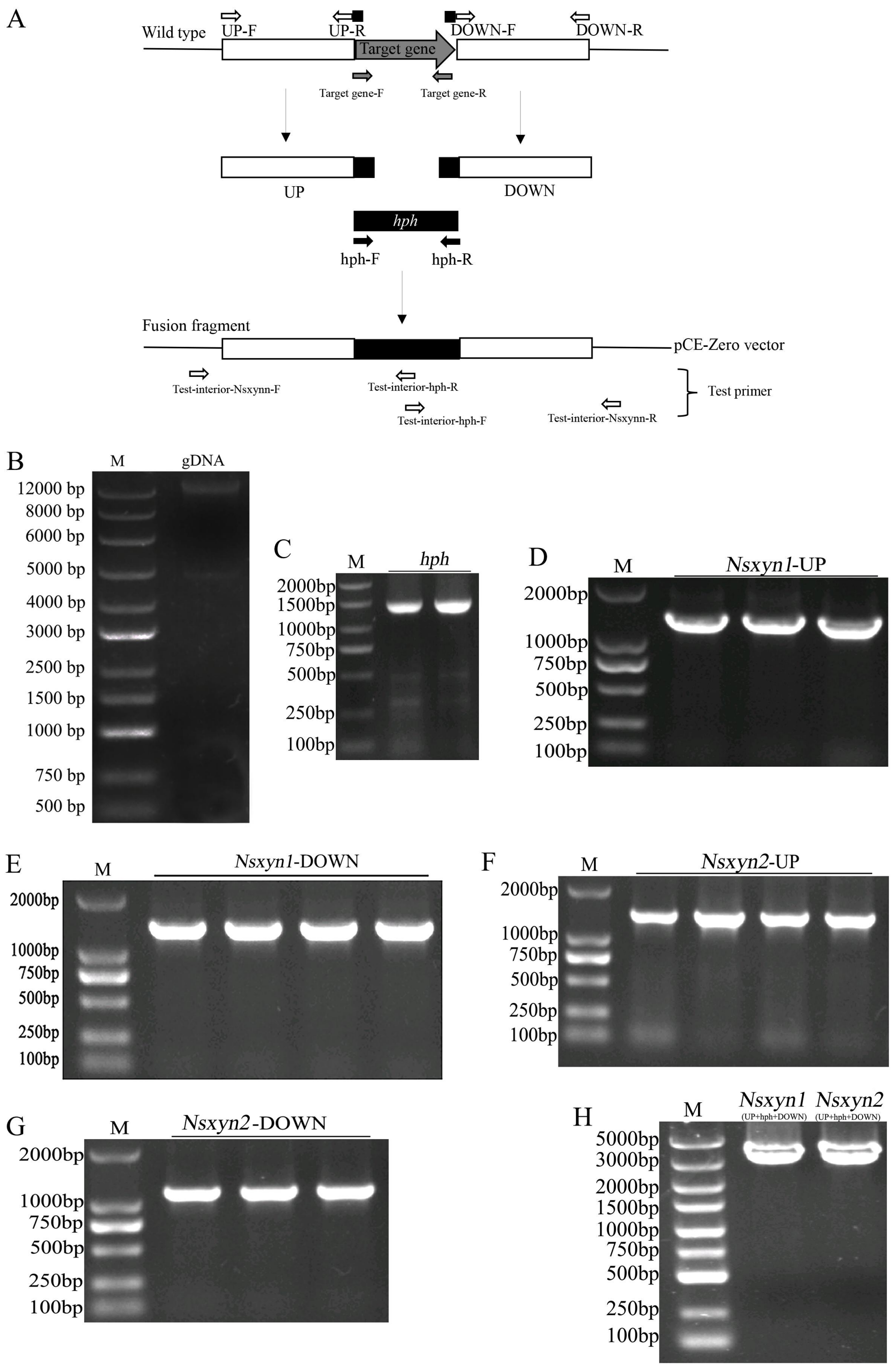

Supplementary Figure 5. The schematic diagram of gene knockout (**A**) and construction of *Nsxyn1* and *Nsxyn2* gene knockout boxes (**B-H**). Detection of gDNA extraction results (**B**), hygromycin fragment (**C**), upstream homology arm of *Nsxyn1* (**D**), downstream homology arm of *Nsxyn1* (**E**), upstream homology arm of *Nsxyn1* (**F**), downstream homology arm of *Nsxyn1* (**G**), and knockout cassette of *Nsxyn1* and *Nsxyn2*. M: DNA marker.

1.6 Supplementary Figure 6

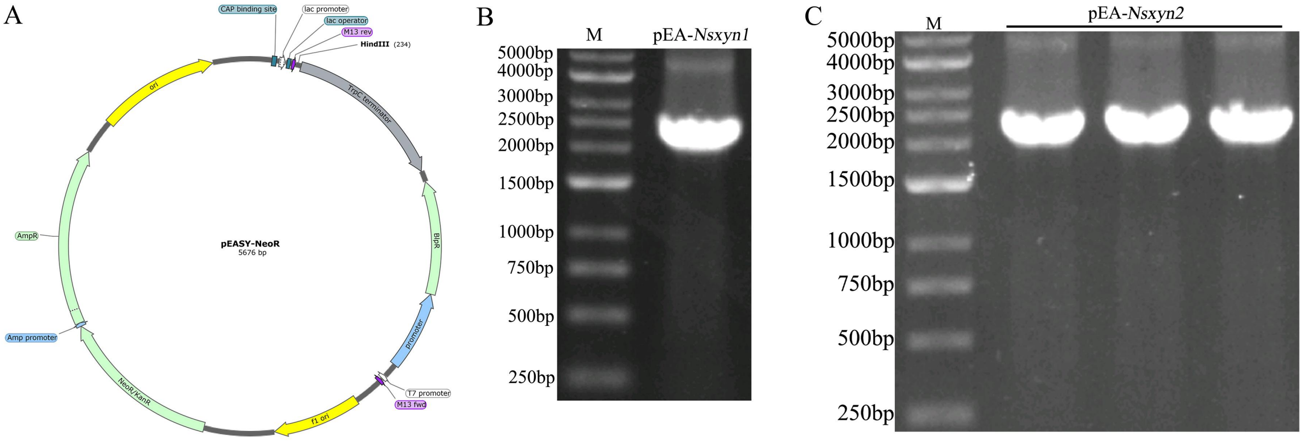

Supplementary Figure 6. Plasmid map of pEASY-NeoR and the identified results of complement vector construction. (**A**) Plasmid map of pEASY-NeoR. (**B-C**) The identified results of complement vector construction of *Nsxyn1* and *Nsxyn2*, respectively. M: DNA marker.

1.7 Supplementary Figure 7

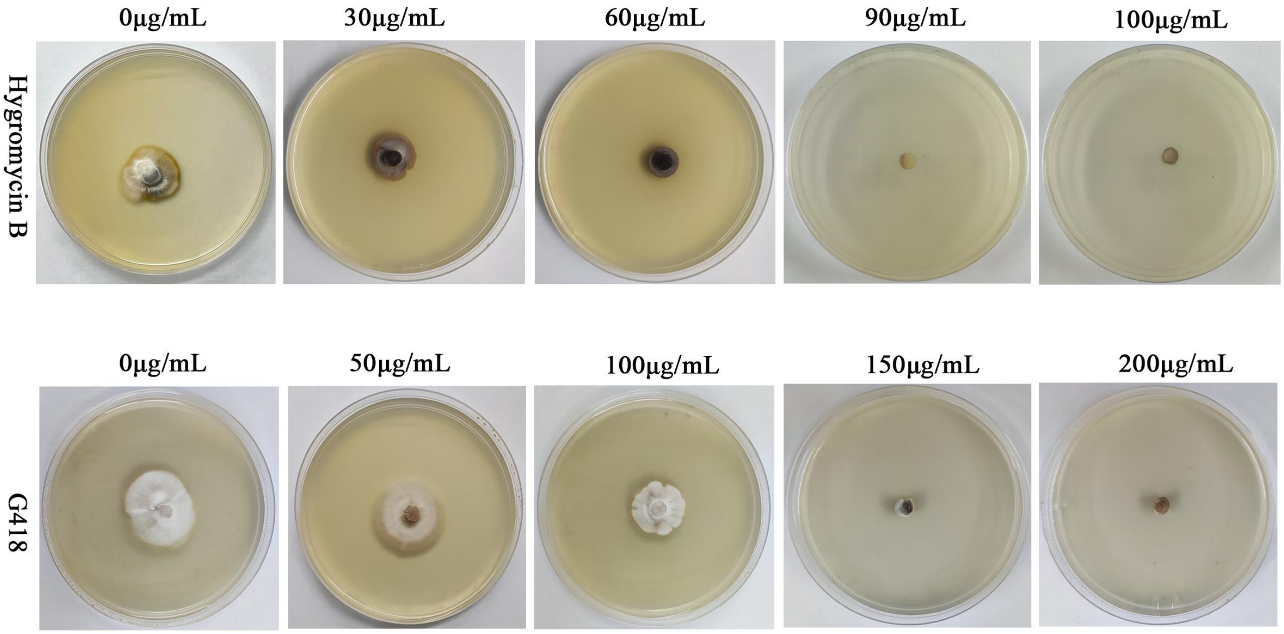

Supplementary Figure 7. The hyg and G418 antibiotic concentration screening.

1.8 Supplementary Figure 8

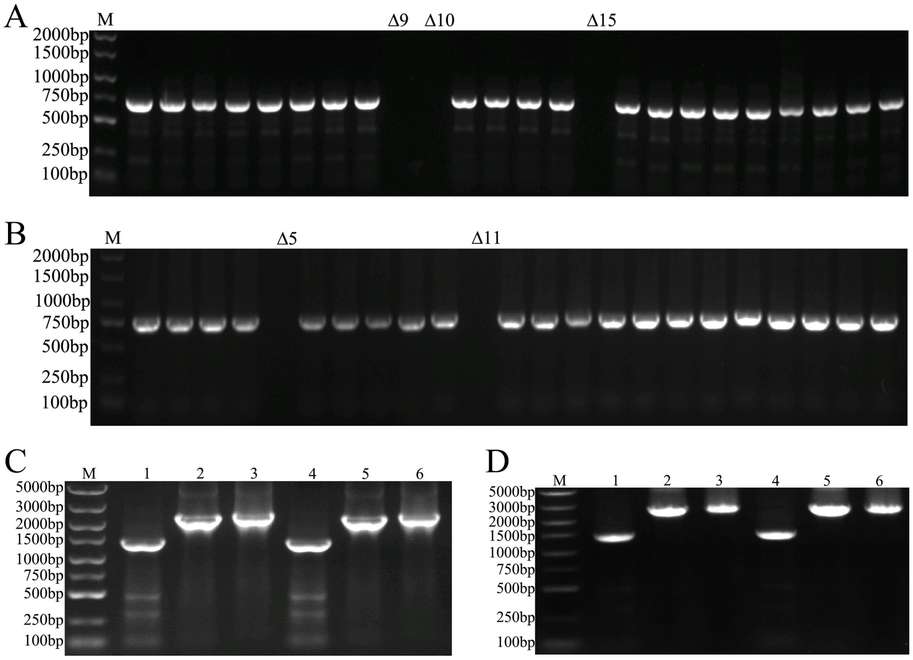

Supplementary Figure 8. PCR verification of knockout transformants. (**A**) Transformants of pCE-*Nsxyn1*. (**B**) Transformants of pCE-*Nsxyn2*. (**C**) pCE-*Nsxyn1*-Δ10. 1: hygromycin fragment detection; 2: upstream fragment detection; 3: downstream fragment detection. (**D**) pCE-*Nsxyn2*-Δ5, 1: hygromycin fragment detection; 2: upstream fragmentdetection; 3: downstream detection. M: DNA marker.

1.9 Supplementary Figure 9

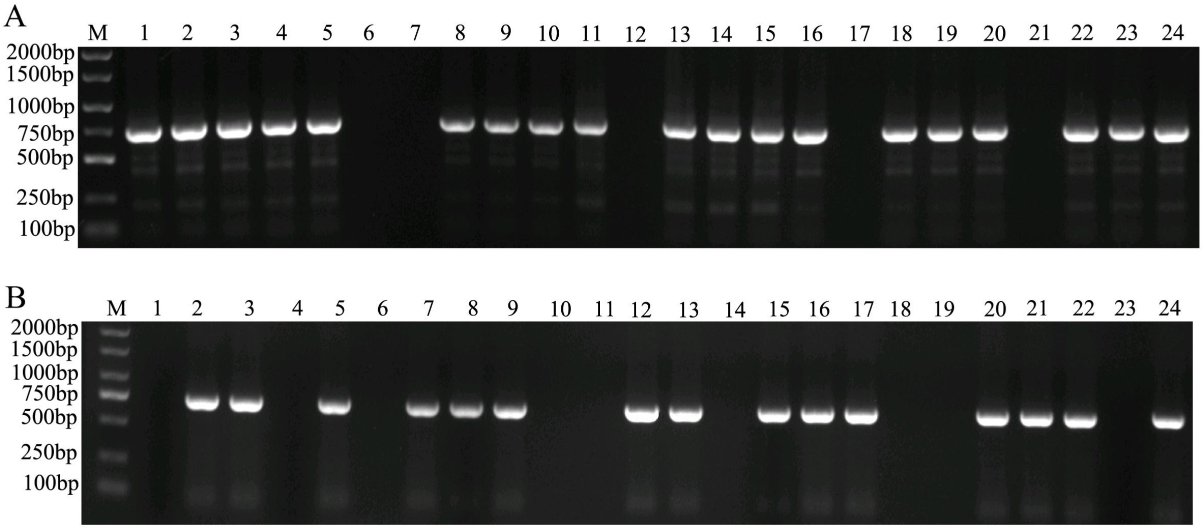

Supplementary Figure 9. PCR verification of the complementary transformants. (**A**) pEASY-*Nsxyn1* complements transformants. (**B**) pEASY-*Nsxyn2* complementary transformants. M: DNA marker.
